# Gravettian cranial morphology and human group affinities during the European Upper Palaeolithic

**DOI:** 10.1038/s41598-020-78841-x

**Published:** 2020-12-14

**Authors:** Aurélien Mounier, Yann Heuzé, Mathilde Samsel, Sergey Vasilyev, Laurent Klaric, Sébastien Villotte

**Affiliations:** 1 Histoire Naturelle de l’Homme Préhistorique (HNHP, UMR 7194), MNHN/CNRS/UPVD, Musée de l’Homme, 17 place du Trocadéro et du 11 novembre, 75016 Paris, France; 2 Leverhulme Centre for Human Evolutionary Studies, Department of Archaeology, University of Cambridge, Fitzwilliam Street, Cambridge, CB2 1QH United Kingdom; 3 UMR 5199 PACEA, CNRS-Université de Bordeaux, Bâtiment B8, Allée Geoffroy Saint Hilaire, Pessac, 33615 France; 4 Institute of Ethnology and Anthropology RAS, Leninsky pr. 32a, Moscow, Russian Federation 119991; 5 CNRS, UMR-7055 PréTech, MSH Mondes, 21 allée de l’Université, 92023 Nanterre, France

**Keywords:** Anthropology, Archaeology, Biological anthropology

## Abstract

Archaeologically defined Upper Palaeolithic (UP, 45,000–10,000 years ago) “cultures” are often used as proxies to designate fossil populations. While recent genomic studies have partly clarified the complex relationship between European UP “cultures” and past population dynamics, they leave open numerous questions regarding the biological characterization of these human groups, especially regarding the Mid-UP period (MUP, 33,000–24,000 years ago), which encompasses a pan-European cultural mosaic (Gravettian) with several regional facies. Here, we analyse a large database of well-dated and well-preserved UP crania, including MUP specimens from South-West France (SWF) and Moravia, using 3D geometric morphometrics to test for human group affinities. Our results show that the Gravettian makers from these two regions form a remarkably phenetically homogeneous sample which is different from, and more homogeneous than, the Late UP sample. Those results are congruent with genomic studies indicating a genetic continuity within the Gravettian manufacturers and a discontinuity marked by the Last Glacial Maximum (LGM). Moreover, our study expands the geographical range of the MUP phenetic continuity to SWF, for which aDNA data are scarce, and clarifies the post-LGM European population structure in SWF, with a possible dual ancestry stemming from different LGM refugia.

## Introduction

Archaeologically defined Upper Palaeolithic (UP, 45,000–10,000 years ago) cultural taxonomic units (“techno-complexes” or “archaeological cultures”) are often used as proxies to designate fossil populations. During the last 100 years, changes in the UP cultural archaeological record have often been scrutinized to debate about population extinctions, migrations, replacements, etc. (for recent controversies and discussions about past models see^1–7^). In the last decade, genomic studies on ancient human DNA (i.e. aDNA) have increased drastically our understanding of the complex relationships between UP techno-complexes and European past population dynamics (e.g.^8^). This is particularly true for the Gravettian techno-complex, during the Mid-UP period (MUP, 33,000–24,000 years ago). The large distribution of the Gravettian techno-complex across the continent has led to the concept of a pan European cultural mosaic, with several identified facies evolving regionally^9–13^. These smaller identified entities often present similarities, but only at certain times and without forming a homogeneous unit on a continental scale for 9,000 years^7,14,15^. Thus, the Gravettian, from a theoretical point of view, can be described as a “vast meta-culture composed by a patchwork of several distinct typo-technological traditions spread over the European continent”^15^.

Ancient DNA studies indicate underlying genetic continuity between manufacturers of MUP industries from Czech Republic, Ukraine, Belgium, and possibly Southern Italy (genetically identified as the Věstonice cluster^16,17^), and a relative proximity between these individuals and some earlier specimens attributed to the Early UP (i.e. EUP, ~ 45,000–33,000 years ago^17–19^). These results have led to the hypothesis of an East European founder population ~ 36,000 years ago from which Gravettian-related groups and industries would have dispersed^16^. The Věstonice ancestry is not seen any more after the Last Glacial Maximum (i.e. LGM ≈ GS3, ~ 26,500–20,000 years ago^20,21^), in the makers of Late UP (i.e. LUP ~ 20,000–10,000 years ago) industries^17,19,22^ such as Magdalenian and Epigravettian.

If the hypothesis of a spread of the Gravettian techno-complex related to migrations of people with their equipment and traditions may stand for some areas in Central or Eastern Europe, more complex evolutionary mechanisms have likely played a role in the genetic structuration of the MUP local populations in Western part of the continent. On the one hand, in several Western European areas (e.g. Belgium, Pyrenean South West France, Cantabria North of Spain), typotechonological data do not support the notion of the Gravettian being an “intrusive culture” that would have simply replaced EUP ones^23–25^. On the other hand, the genetic history of Western European MUP local populations remains poorly understood. This is especially the case for the South West of France (SWF), an area that yielded one of the richest corpus of MUP human fossils chronologically distributed through the whole period^26^. In this region, Gravettian is characterized by a succession of phases (Early, Middle, Late and Final) showing a diversity of lithic projectile points and implements and a no less remarkable diversity of *chaines opératoires* of blades and bladelets production^10,27,28^. In SWF, Gravettian art is also prominent, with several ornamented caves with stylized animal figures carved or painted and negative hands^29^. Several sites in SWF also delivered female figures carved on cave-wall (Cussac) or fallen rocks (Laussel) or made of ivory (Brassempouy, Lespugue) or soft stones (Tursac, Sireuil), that are often stylistically compared to the ones known in the rest of Europe (see for instance^30,31^). Gravettian in SWF is also characterized by complex funerary behaviours implying deliberate commingling of the remains of several individuals, unique in the MUP mortuary landscape^32–34^. To date, no nuclear DNA has been extracted from SWF MUP human remains, but a mitochondrial DNA (i.e. mtDNA) sequence belonging to the M haplogroup was published for the La Rochette individual dated at ~ 27,500 years ago. This haplogroup has also been identified in four EUP specimens from Goyet (Belgium) and Bacho Kiro (Bulgaria) and in one Italian MUP fossil from Ostuni (Italy)^17,19,35,36^, but has not been found in EUP or MUP individuals from Central Europe. In this context, the genetic history of the MUP groups remains unclear, and the biological affinities that existed between the SWF MUP local population and other MUP groups are not properly understood.

**Figure 1:**
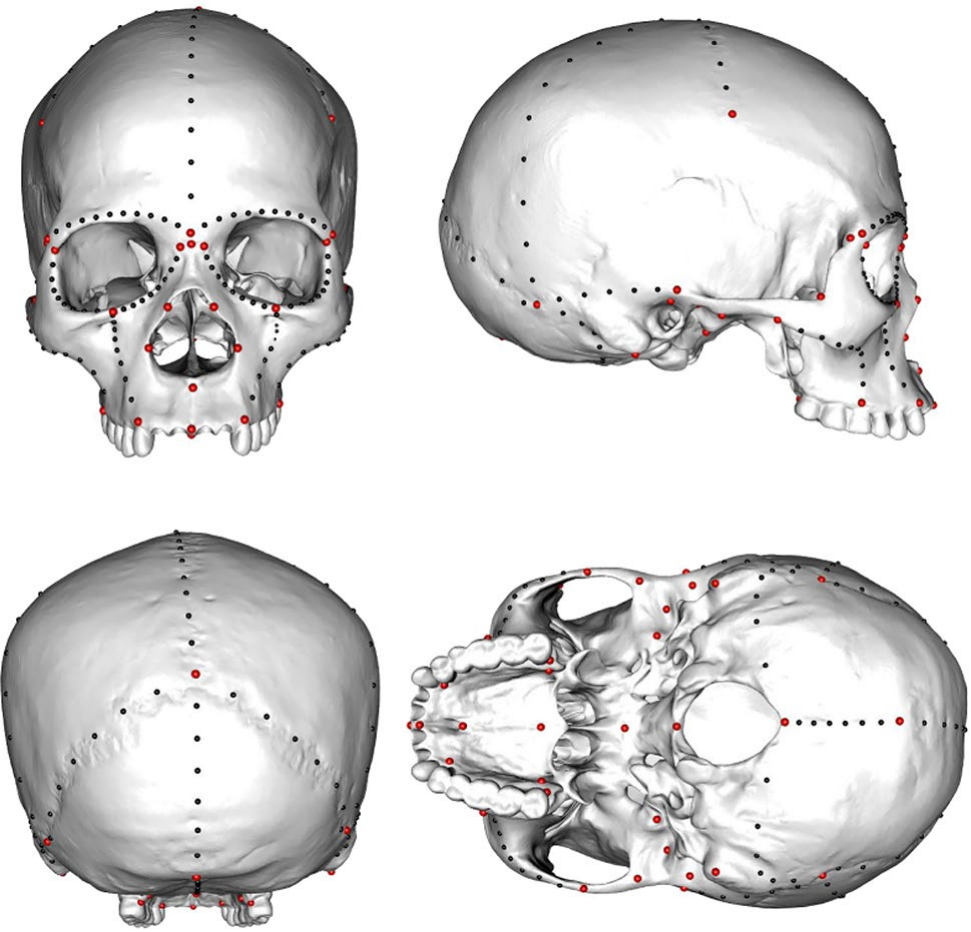
Description of the landmarks (red) and semi-landmarks (black) used in the analyses.

The present study addresses this issue by using 3D geometric morphometrics on 26 well-dated and well-preserved UP crania, including four MUP specimens from SWF and seven MUP fossils from Moravia (Table 1). Geometric morphometrics provides a robust set of methodological tools to decipher phenetic affinities between isolated fossil specimens or paleodemes (see^37^). It has been shown that patterns of cranial morphology largely reflect genetic distances among human populations and that this morphology is evolving largely neutrally^38–41^. Geometric morphometrics have been used extensively in palaeoanthropological studies in the past two decades, more specifically to address early periods of human evolution at a time when different human species coexisted (see for instance^42–45^), but to our knowledge, they have never been used to discuss European UP human group affinities. Here, we apply geometric morphometrics using cranial 3D landmarks and semi-landmarks (Fig. 1) to characterise and quantify:

1. The morphological affinities between the EUP, MUP and LUP samples;
2. The morphological affinities between the SWF MUP individuals and the other MUP sample; and
3. The degree of morphological homogeneity of the entire MUP and LUP samples.

**Table 1:** Fossil specimens included in the study.

Sample	Site	Specimens	Sex	Labels
EUP (37–34,000 years ago)	Kostënki-Borshchyovo, Russia	Kostënki 14*	M	Ko14
	Sungir’, Russia	Sungir’ 1*	M	Sun
	Mladeč, Czec Republic	Mladeč 1*	F	Ml1
	Peştera Muierii, Romania	Muierii 1*	F	Mui
MUPswf (32–27,000 years ago)	Cro-Magnon, France	**Cro-Magnon 1**	M	CM1
	Cro-Magnon, France	**Cro-Magnon 2**	F?	CM2
	Cussac, France	**Cussac L2A**	M	Cus
	Abri-Pataud, France	**Abri Pataud 1**	F	AP1
MUPmor (31–30,000 years ago)	Dolní Věstonice I, Czech Republic	**Dolní Věstonice 3**	F	DV3
	Dolní Věstonice II, Czech Republic	Dolní Věstonice 13*	M	DV13
	Dolní Věstonice II, Czech Republic	Dolní Věstonice 14*	M	DV14
	Dolní Věstonice II, Czech Republic	Dolní Věstonice15*	M	DV15
	Dolní Věstonice II, Czech Republic	Dolní Věstonice 16*	M	DV16
	Předmostí, Czech Republic	Předmostí 3	M	Pr3
	Předmostí, Czech Republic	Předmostí 4	F	Pr4
LUPswf (19–18,000 years ago)	Abri du Cap Blanc, France	Cap Blanc	F	CB
	Chancelade, France	Chancelade 1*	M?	Cha
	Abri Lafaye, France	Lafaye 24*	F	Laf
	Grotte du Placard, France	Le Placard 142*	F	LP
	Saint-Germain La Rivière, France	Saint-Germain La Rivière 4*	F	SGLR
LUPita (15–11,000 years ago)	Caverna delle Arene Candide, Italy	**Arene Candide 2**	M	AC2
	Caverna delle Arene Candide, Italy	Arene Candide 3*	M	AC3
	Caverna delle Arene Candide, Italy	**Arene Candide 4**	M	AC4
	Grotta di San Teodoro, Italy	San Teodoro 1*	F?	ST1
	Grotta di San Teodoro, Italy	**San Teodoro 2**	M	ST2
	Riparo di Villabruna, Italy	Villabruna 1*	M	Vil

More information on the sample can be found in the online supplementary material (Supplementary Table S1). Specimens in bold: data from the original fossils.

*Direct dating of the skeleton.

## Results

### Morphological affinities between EUP, MUP and LUP samples

**Figure 2:**
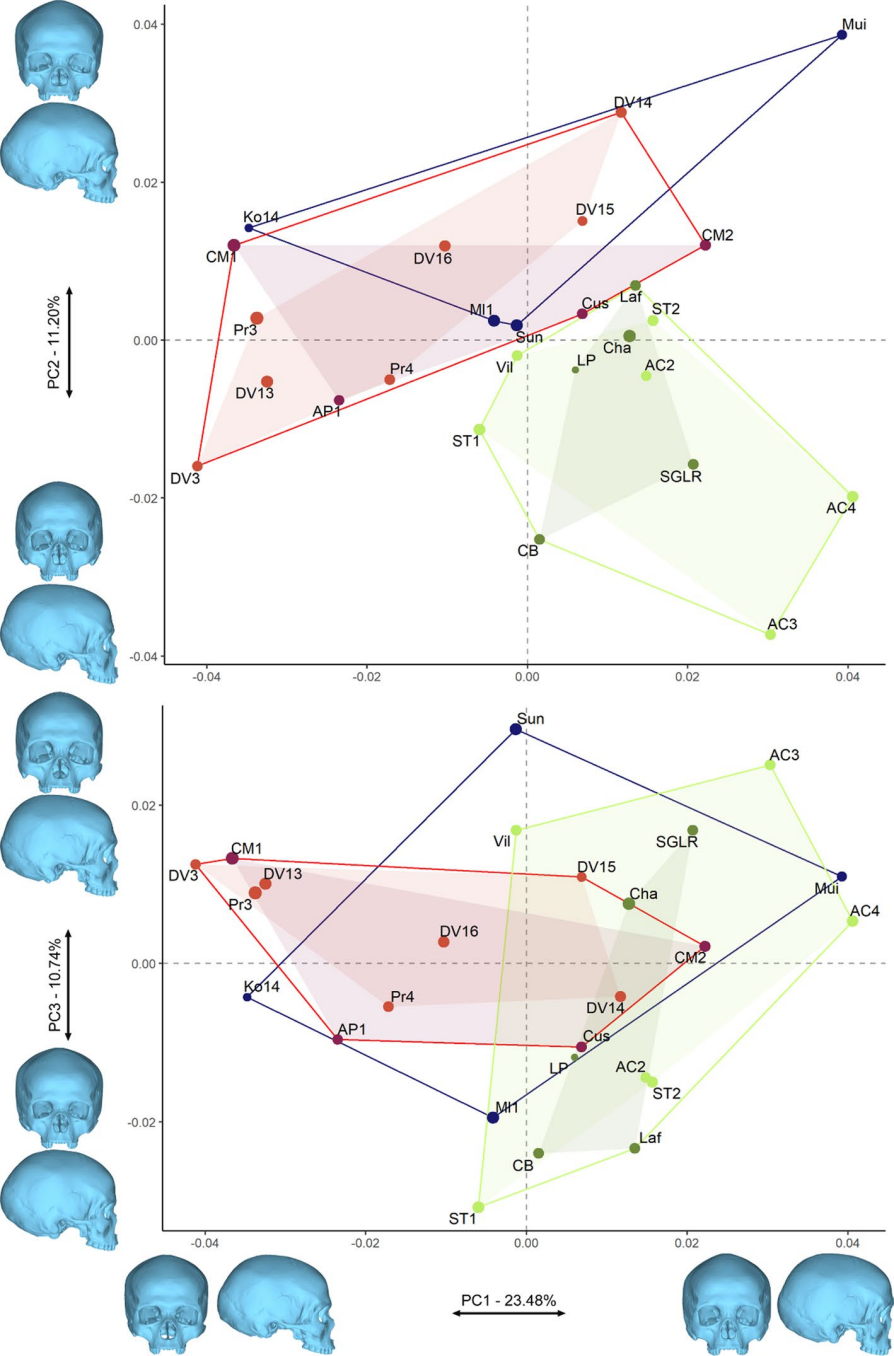
PC1, 2 and 3 representing 45.43% of the variation of the morphospace. Convex hulls show the morphological variation of the specimens (dark blue: EUP; red: MUP, MUPswf in violet and MUPmor in red; green: LUP, dark green LUPswf and light green LUPita). The size of the data points reflects the centroid size of the specimens. PC1 and 2 separates the MUP and EUP samples from the LUP specimens.

Figure 2 shows the morphospace defined by PCs 1 to 3 (45.43% of the total variation). None of the first three PCs alone successfully discriminate one of the three fossil samples, however, and as highlighted by the convex hulls, The EUP and MUP samples do not overlap with the LUP group in the morphospace defined by PC1 and 2 only (34.69%, Fig. 2). The EUP and MUP specimens tend to have longer brain case with a small occipital bun, along with a more projecting face than the LUP individuals. While there are no outliers within the full fossil sample (Supplementary Fig. S6a), DV3 appears to be an outlier within the MUP sample (Supplementary Fig. S6c). This may be due to the mild cranial deformation related to a facial injury on the specimen^46^. PCA results are confirmed by the Mahalanobis and Procrustes distances between the different samples. The cranial shape of the MUP and EUP specimens cannot be statistically distinguished, while that of the LUP specimens statistically differs from the MUP sample (Table 2).

**Table 2:** Mahalanobis and Procrustes distances between the samples calculated from the aligned 3D coordinates and associated p-values from permutation tests (10,000 permutations rounds).

		EUP	MUP	LUP
		*p*		
EUP	Maha. dist	–	0.997	0.091
MUP		2.2534	–	**< 0.0001**
LUP		3.0322	**3.1609**	–
EUP	Proc. dist	–	0.445	0.082
MUP		0.0270	–	**0.0003**
LUP		0.0337	**0.0335**	–

Small specimens tend to have larger braincases and smaller faces than large specimens (Supplementary Fig. S7e). Nevertheless, allometry does not explain the patterns described by the morphospace as demonstrated by the non-significant results of the linear regressions presented in Table S3 in the supplementary online material (Supplementary Table S3 and Fig. S7).

**Figure 3:**
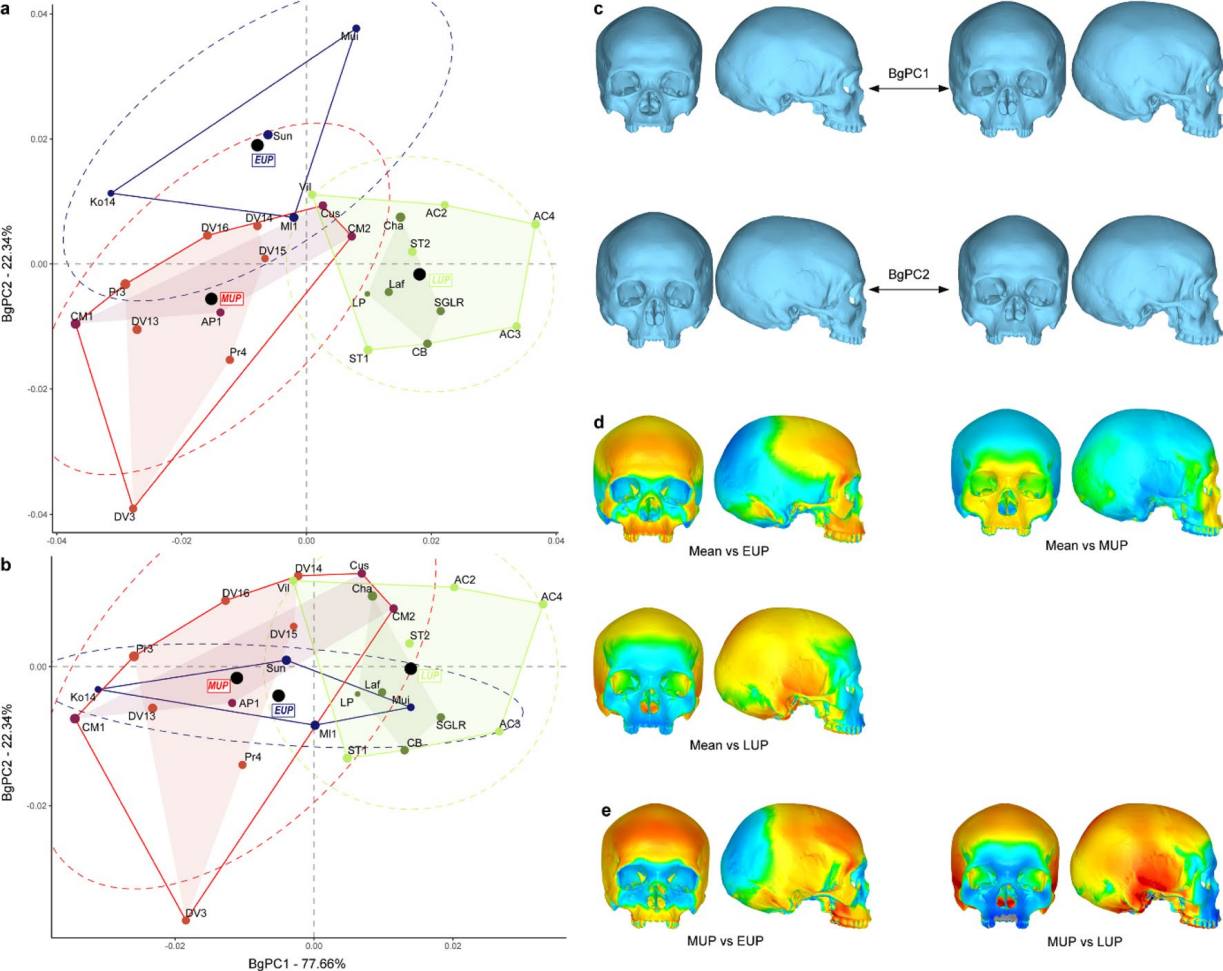
Between-group PCA discriminating the EUP, MUP and LUP groups. (**a**) morphospace of the bgPCA; (**b**) morphospace after cross-validation; (**c**) shape changes for bgPC1 and bgPC2; (**d**) surface deviation spectrum showing the shape difference between the mean shape of the entire sample versus the mean shape of each group; (**e**) surface deviation spectrum showing the shape difference between MUP and the two other fossil groups. For each group, black circles indicate the position of the centre, and the dotted ellipses represent the 90% confidence intervals. The size of the data points reflects the centroid size of the specimens.

The Fig. 3 presents the morphospace of the bgPCA when testing for the existence of three different groups (EUP, MUP and LUP). While the first bgPC (77.66% of the variation, see Supplementary Table S4) discriminates between EUP/MUP and LUP specimens, bgPC2 (22.34%) seems to discriminate between EUP and MUP/LUP specimens (Fig. 3a). However, after cross-validation the discrimination between EUP and MUP/LUP disappears while the discrimination between LUP and the two other groups remains (Fig. 3b). This trend is confirmed by the Euclidean distances between groups (Table 3) which yields similar results to the Mahalanobis and Procrustes distances from Table 2. EUP and MUP are not different shape wise, while both samples appear different from the LUP specimens, although difference between LUP and EUP is not significant (Table 3). Regarding shape difference, the MUP and EUP specimens present on average a more projecting face and less globular braincase when compared to the mean shape. EUP specimens have nevertheless a more projecting frontal bone while facial projection is mainly expressed in the lateral expansion of the zygomatic and in the anterior projection of the lower maxilla. LUP specimens show a more rounded braincase and a more retracted upper face (Fig. 3d). Using the mean MUP shape as a reference in the comparison with the other group average shapes further highlights the identified patterns. Compared to the MUP, the EUP average shape shows an expansion of the frontal and lower maxilla, while the LUP mean shape shows a global expansion of the braincase, especially at the temporal, and a strong retraction of the face (Fig. 3e).

**Table 3:** Euclidean distances between the group averages calculated from the 15 first PCs. P-values of pairwise sample differences based on permutation testing.

		EUP	MUP	LUP
		*p*		
EUP	Euc. dist	–	0.505	0.093
MUP		0.0258	–	**0.0003**
LUP		0.0333	**0.0336**	–

### Morphological affinities between MUP subsamples

**Figure 4:**
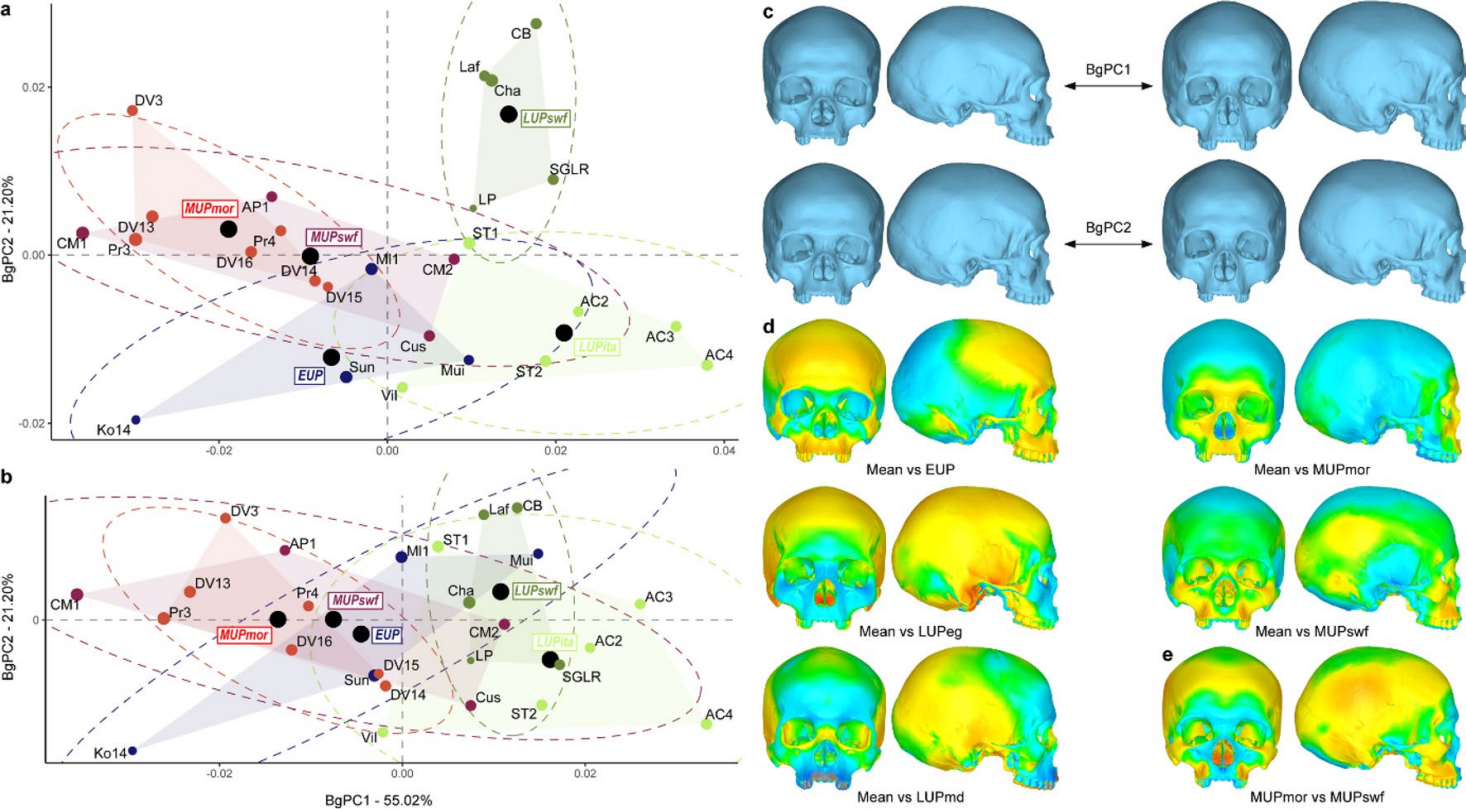
Between-group PCA discriminating the EUP, MUPswf, MUPmor, LUPswf and LUPita samples. (**a**) morphospace of the bgPCA; (**b**) morphospace after cross-validation; (**c**) shape changes for bgPC1 and bgPC2; (**d**) surface deviation spectrum showing the shape difference between the shape difference between the mean shape of the entire sample versus the mean shape of each group. For each group, black circles indicate the position of the centre, and the dotted ellipses represent the 90% confidence intervals. The size of the data points reflects the centroid size of the specimens.

A second bgPCA was run separating the MUP and LUP specimens according to their geographical location for the MUP fossils (i.e. MUPswf and MUPmor) and to their geographical location and chrono-cultural attribution for the LUP (i.e. LUPswf and LUPita). The results displayed on Fig. 4 identify a similar discrimination pattern as observed in the previous bgPCA with bgPC1 (55.02% of the variation, see Supplementary Table S5) separating the LUPswf and LUPita from the rest of the sample. None of the bgPC successfully separates the EUP from the MUP specimens and the MUPswf and MUPmor groups overlap completely (Fig. 4a). BgPC2 (21.20% of the variation, see Supplementary Table S5) separates the LUPswf and the LUPita specimens. However, after cross-validation, the discrimination between the LUP subgroups disappears (Fig. 4b). The shape changes associated to this morphospace are similar to the previous bgPCA (Fig. 4c). This is also the case for the surface deviation spectrum expressing the differences in shape between the average shape of the entire sample and the average shape of each group (Fig. 4d). More interesting is the difference we can observe between the MUP and LUP subgroups. The LUPita shape is similar to the overall shape characteristic of the LUP specimens (i.e. more globular braincase and retracted upper face) while it is different for the LUPswf: less projecting frontal and brow-ridges and less retracted upper face (Fig. 4d). Similarly, the MUPmor specimens conform to the overall shape of the MUP group (i.e. more projecting face, less globular calvarium) while the MUPswf specimens present a slightly different pattern showing an expansion of the parietal and of the posterior temporal at the asterion level, the lower part of the maxilla is also more projecting laterally (Fig. 4d). These differences are highlighted by the direct comparison of the mean MUPmor to the mean MUPswf shape (Fig. 4e).

The Mahalanobis, Procrustes and Euclidean distances (Tables 4 and 5) confirm the identified tendencies: the MUPswf and MUPmor fossil samples are not significantly different, and cannot be differentiated from the EUP specimens. However, the MUPmor fossil sample is significantly different from the LUP groups, while the MUPswf specimens appear to be closer in shape to the LUP specimens – especially those associated to the Magdalenian techno-complex (Mahalanobis, Procrustes and Euclidean distances, respectively 3.2599, 0.0336 and 0.0329), than the MUPmor (Mahalanobis, Procrustes and Euclidean distances, respectively 3.3704, 0.0417 and 0.0387).

**Table 4:** Mahalanobis distances between the groups calculated from the aligned 3D coordinates and associated p-values from permutation tests (10,000 permutations rounds).

		EUP	MUPmor	MUPswf	LUPswf	LUPeg
		***p***				
EUP	Maha. dist	–	0.961	0.998	0.811	0.363
MUPmor		2.5224	–	0.964	**0.002**	**0.004**
MUPswf		1.9694	2.4630	–	0.14	**0.037**
LUPswf		2.9622	**3.3704**	3.2599	–	0.694
LUPita		3.0234	**3.2828**	**3.4343**	2.7184	–
EUP	Proc. dist	–	0.369	0.938	0.160	0.149
MUPmor		0.0311	–	0.723	**0.005**	**0.002**
MUPswf		0.0265	0.0248	–	0.203	0.099
LUPswf		0.0372	**0.0417**	0.0336	–	0.180
LUPita		0.0367	**0.0389**	0.0366	0.0302	–

**Table 5:** Euclidean distances between the group averages calculated from the 15 first PCs. P-values of pairwise group differences based on permutation testing.

		EUP	MUPmor	MUPswf	LUPswf	LUPita
		*p*				
EUP	Euc. dist	–	0.333	0.907	0.123	0.110
MUPmor		0.0302	–	0.794	**0.020**	**0.003**
MUPswf		0.0244	0.0238	–	0.307	0.110
LUPswf		0.0371	**0.0387**	0.0329	–	0.030
LUPita		0.0360	**0.0418**	0.0363	0.280	–

### MUP within-group morphological variance

**Figure 5:**
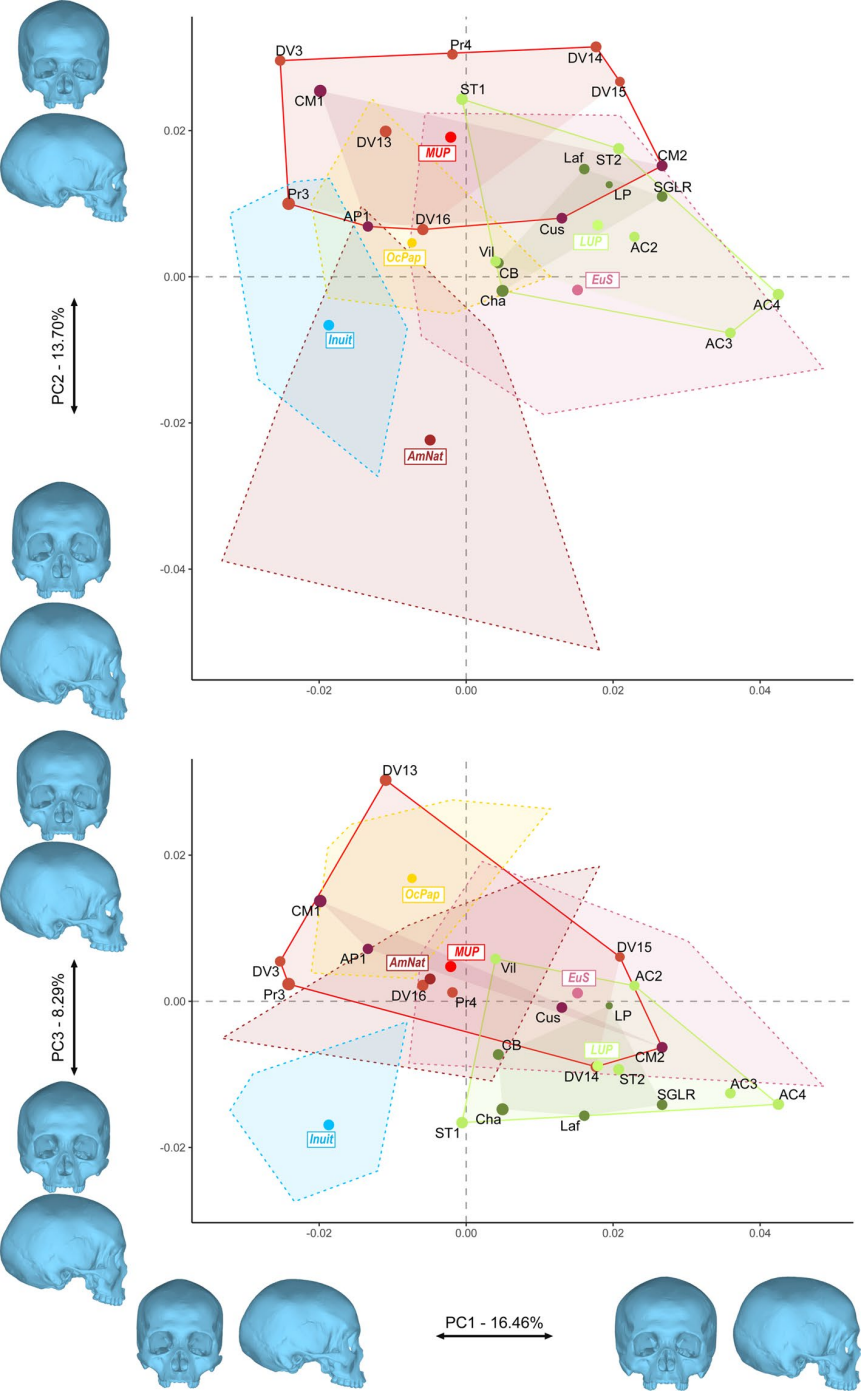
PC1, 2 and 3 representing 38.45% of the variation of the morphospace of the MUP, LUP and extant human samples. Convex hulls show the morphological variation of the specimens (red: MUP, MUPswf in violet and MUPmor in red; green: LUP, dark green LUPswf and light green LUPita; light blue: Inuit; yellow: Papuans; brown: Native North Americans; and pink: South Europeans). Extant modern human samples are only represented through their convex hull and the average shape of the sample. The size of the data points reflects the centroid size of the specimens and group means.

To understand the cohesiveness of the MUP sample we ran an additional Generalized Procrustes Analysis^47,48^ (i.e. GPA, see “Methods”), using an extended sample including specimens from four extant human groups (i.e. North America –AmNat, South Europe –EuS, Greenland –Inuit, and Papua New Guinea—OcPap, see Supplementary Table S1), while the EUP specimens were excluded (see, Methods). The specimens were aligned and based on the Procrustes residuals, the full sample had three outliers (i.e. two Inuit and the AC3 specimen, Fig. S3). However, there was no outlier within each group to the exception of DV3 which, as noted earlier, was slightly outside of the expected range of variation of the MUP group (see, Supplementary Fig. S8). The analysis was run with and without DV3 (Tables 6 and 7). There was no marked size difference between the groups (Supplementary Fig. S9), and size did not have a significant impact on shape on PC1 to PC6 (Supplementary Table S6, Fig. S10) which were used to run the analysis. The discrimination of the different groups was poor on the morphospace defined by PCs1 to 3 (Fig. 5), but for the AmNat specimens which were mostly separated on PC2 (13.70% of the variation, see Supplementary Table S6). In terms of spread of the different groups in the morphospace, the AmNat specimens occupied the largest part of the morphospace, followed by the EuS, MUP and LUP samples. The Inuit and OcPap samples, as expected, appeared less variable.

To better appreciate the observed variation within the different groups, we tested for the proportionality of the covariance matrices and computed the ratios of generalized variances of the groups (Tables 6 and 7). The extant modern human samples followed the expected patterns: the AmNat group was the most variable, followed by the EuS one (which presented about 11% of the variation observed within the AmNat sample); the OcPap group was much less variable (with over 200 times more variation within the AmNat sample). The MUP specimens presented less variation than the broadly defined geographical AmNat sample as they showed only 22% of the variation expressed within the AmNat group. They were nevertheless more variable than the EuS sample, showing almost twice as much variation. When compared with the ethnically defined sample, the MUP specimens appeared more variable than the Inuit (i.e. about six times the Inuit’ variation). Finally, the LUP sample’s covariance matrix presented three times more variation than the MUP individuals and were about as variable as the AmNat sample (i.e. ~ 70% of the AmNat within group variation, see Table 6). When excluding DV3, the MUP within-group variation was reduced but we observed a similar within-group variation pattern to the exception of the comparison of the MUP and EuS sample (Table 7). The MUP sample was then less variable than the EuS (i.e. ~ 75% of the variation observed within the EuS group).

**Table 6:** Covariance matrices ML proportionality test and ratios of generalized variances between groups based on the entire sample.

	AmNat	EuS	Inuits	OcPap	MUP	LUP
**Prop.test**						
AmNat (N = 11)	–					
EuS (N = 11)	**0.002**	–				
Inuits (N = 11)	0.261	0.053	–			
OcPap (N = 11)	**0.04**	0.056	0.062	–		
MUP (N = 11)	**0.005**	**0.001**	**0.002**	0.254	–	
LUP (N = 11)	**0.037**	**0.008**	0.489	0.061	**0.011**	–
**Ratios**						
AmNat (N = 11)	–	**8.82**	29.08	**247.34**	**4.37**	**1.43**
EuS (N = 11)	**0.11**	–	3.298	28.05	**0.51**	**0.16**
Inuits (N = 11)	0.03	0.30	–	8.50	**0.16**	0.05
OcPap (N = 11)	**0.00**	0.036	0.12	–	0.02	0.01
MUP (N = 11)	**0.22**	**1.94**	**6.41**	54.51	–	**0.32**
LUP (N = 11)	**0.70**	**6.15**	20.29	172.58	**3.17**	–

Significant results (*p* < 0.05) are shown in bold and are the only results discussed in the text.

**Table 7:** Covariance matrices ML proportionality test and ratios of generalized variances between groups after exclusion of the outlier DV3 (see, Supplementary Fig. S8).

	**AmNat**	**EuS**	**Inuits**	**OcPap**	**MUP**	**LUP**
Prop.test						
AmNat (N = 11)	–					
EuS (N = 11)	**0.023**	–				
Inuits (N = 11)	0.272	0.066	–			
OcPap (N = 11)	**0.002**	**0.004**	**0.004**	–		
MUP (N = 10)	**0.032**	**0.006**	**0.007**	0.143	–	
LUP (N = 11)	**0.038**	**0.009**	0.503	**0.033**	**0.032**	–
Ratios						
AmNat (N = 11)	–	**6.37**	26.58	**320.20**	**8.46**	**1.16**
EuS (N = 11)	**0.16**	–	4.17	**50.27**	**1.33**	**0.18**
Inuits (N = 11)	0.04	0.24	–	**12.04**	**0.32**	0.04
OcPap (N = 11)	**0.00**	**0.02**	**0.08**	–	0.03	**0.00**
MUP (N = 10)	**0.12**	**0.75**	**3.14**	37.84	–	**0.14**
LUP (N = 11)	**0.86**	**5.50**	22.95	**276.44**	**7.30**	–

Significant results (*p* < 0.05) are shown in bold and are the only results discussed in the text.

In sum, the MUP sample appeared to be less variable or presenting a similar within group variation than the geographically broadly defined groups (i.e. respectively AmNat and Eus) but showed more variation than the ethnically defined samples (i.e. Inuit and OcPap). The MUP sample also seemed to be more homogeneous than the LUP sample which was formed by two different techno-complexes: Epigravettian and Magdalenian.

## Discussion

The present study is the first geometric morphometric analysis focusing on cranial morphological affinities between European UP local populations. No significant differences in shape between EUP and MUP individuals were found confirming previous genomic^17–19^ and cranial osteometric^49^ studies. The close morphological affinity between the two samples may have been partly influenced by the very limited number of available EUP specimens, but it could also reflect true human group affinities. For instance, the phenetic similarities between the EUP specimens Kostënki 14 (likely related to the Early Aurignacian techno-complex) and Sungir’ 1 (associated to the Streletskian, a techno-complex that clearly differs from the Aurignacian and the Gravettian), and the MUP morphotype in our analyses, concur with recent genomic results showing genetic proximity between these fossils and those from the Věstonice cluster^17,18^.

Our analyses significantly distinguish MUP and LUP samples. This result was expected considering the disappearance of Věstonice ancestry in post-LGM fossils^19^, and previous anthropological studies on cranial and infracranial morphology have already highlighted the striking morphological differences between these two groups^49–54^. Our results are mostly congruent with previous cranial osteometric analyses which identified most of the differences on the face^49,50^: LUP specimens display on average a less projecting face, shorter minimal frontal breadth and nasal aperture dimensions, and slightly larger orbital breadth (Fig. 3d). Contrary to Churchill et al.^49^, however, LUP crania did not appear smaller than the pre-LGM specimens included in our study, but instead seemed to be more homogeneous regarding size (Supplementary, Fig. S9). Those results, as well as previous ones from genomic and morphological studies, clearly indicate that the UP fossil sample is heterogeneous, thus, it appears essential to avoid pooling MUP and LUP fossils together in future paleoanthropological studies.

Our results highlight cranial morphological differences between MUP and LUP fossils from the same region (SWF), though they are less marked than in other MUP and LUP comparisons (i.e. MUPmor and LUP subsamples, and MUPswf and LUPita). These differences may be related to the suggested population bottleneck during the LGM^19^, which could have caused a strong genetic drift with the fixation of certain morphological characters. Alternatively, this apparent change in cranial morphology may indicate the arrival of new ancestry during the LGM, that would have partly replaced or admixed with the local one. Both hypotheses are not mutually exclusive (see infra).

Ancient DNA studies^17,19,22^ identified two main ancestries during the LUP. The Goyet Q-2 ancestry is predominant in 19,000–15,000-year-old individuals associated with the Magdalenian culture. This genetic ancestry was largely replaced by the ‘Villabruna ancestry’ ~ 14,000 years ago, suggesting migrations or population shifts at that time. None of the LUPswf specimens has been genetically characterized so far, but considering that they are dated to ~ 19, 000 years ago while the LUPita sample dates to the second part of the LUP (15,000–10,000 years ago), significant morphological differences between these two samples would be expected. However, it has been shown recently that Iberian hunter-gatherers, including the El Mirón individual dated to ~ 19,000 years ago, carried dual ancestry from both Villabruna and Goyet Q-2 clusters^22^. Given that South-Western Europe (i.e. the Iberian Peninsula and SWF) was likely a refugium for human groups during the LGM, it seems possible that our LUPswf sample also carries a dual genetic signature similar to that of the El Mirón cluster. The presence of Villabruna-like ancestry in this genetic signature could explain the morphological similarities observed between the LUPswf and the latter LUPita sample. Alternatively, the LUP from SWF may carry a full Villabruna ancestry. This hypothesis implies that both LUPswf and LUPita would have originated from a unique founding event which would have most likely taken place in the Italian peninsula at least ~ 20,000 years ago. Given the high degree of within-sample variation in the LUP sample (i.e. at least thrice more than the one observed within the MUP sample, see Tables 6 and 7), the first scenario appears more likely.

The two geographical MUP samples could not be statistically distinguished as they occupied the same morphospace. This may indicate that the MUPswf sample is associated to the Věstonice cluster, and hence, both human groups may have originated from a single founding event. Alternatively, as EUP fossils could not be easily distinguished from MUP ones based on their morphology (and as it seems to be the case as well for the French EUP frontal bone La Crouzade V^55^), it is also possible that people carrying the Věstonice ancestry and coming from the East have mixed with local populations in SWF France. In any case, the remarkable morphological homogeneity seen for the MUP sample, despite the distance between SWF and Moravia (~ 1200 km) and the time span (over 6000 years), suggests that the two areas did not stay isolated throughout the MUP. Indeed, the gene flow rate appears to have been high enough to prevent local populations from diverging through genetic drift, and both groups may have belonged, at least for some times, to the same large mating networks. These complex processes of population migrations and interactions and the spread of genes (and likely of knowledge and ideas as well) through mating networks reinforce the idea of the Gravettian as a vast meta-culture composed by a patchwork of several regional distinct typo-technological traditions.

## Conclusion

The present study used 3D geometric morphometrics to analyse for the first time the largest well-dated and well-preserved European UP crania sample. This approach offers a unique means to decipher the complex phenetic signals within the European UP fossil sample. The affinity of the different regional UP human groups considered to be the makers of the various meta-cultures composed by a patchwork of several distinct typo-technological traditions have recently been the focus of numerous genomic studies which have paved the way to a better understanding of the complex population structures within European UP human groups^17,19,22^. The main results of the present study show that part of the makers of some facies of the Gravettian meta-culture, the MUP specimens from SWF (Early, Middle and Final Gravettian) and Moravia (Pavlovian), are phenetically homogeneous and could be considered as a widespread unique paleodeme (*sensu*^37^). They share affinities with EUP fossils, but are different from post-LGM LUP specimens, which within group diversity is much more important than what can be observed within the MUP sample. After the LGM, and as suggested by genomic results^19,22^ for the Iberian Peninsula, the human groups in SWF were likely partly replaced and/or admixed with groups coming from a refugium in Southern Italy, comforting the idea that LUP human groups were likely structured around at least two genetic ancestries stemming from different LGM refugia.

## Materials and methods

### Materials

The UP sample was composed of 26 well-preserved crania (i.e. with preserved calvarium and upper face, Supplementary Table S1). The MUP sample (n = 11) was subdivided into two geographic areas: SWF (MUPswf, n = 4, Supplementary Fig. S3) and Moravia (MUPmor, n = 7, Supplementary Fig. S2). The EUP sample was composed of four crania from Russia, Czech Republic and Romania (Supplementary Fig. S1). Eleven LUP crania were used in the analyses, six from Italy (LUPita, Supplementary Fig. S5) and associated to the late Epigravettian techno-complex and five from France (LUPswf, Supplementary Fig. S4) associated to the early phase of the Magdalenian techno-complex. To assess the MUP within-group variation, we used a comparative sample composed of nineteenth century crania drawn from four human groups (n = 11 for each sub-sample to match the sample size of the LUP and MUP samples). The groups were not chosen based on their geographic origin but on the reliability of their identification as discrete entities. Two of those groups are based on large geographic areas (Native North Americans from British Columbia, Ontario, the Midwest and New Mexico -AmNat; Southern Europeans from France, Italy and Malta -EuS, Supplementary Table S1) and two are based on better defined ethnic groups (Greenland Inuit -Inuit; New Guinea Papuans -OcPap, Supplementary Table S1). The morphology of the sample is not being directly investigated, but their morphological variation is used as a model to investigate the within-group variation of the LUP and MUP samples. Additional information on the material can be found in the Supplementary file.

The sample is estimated to be composed of 38.6% female individuals (42.3% for the fossil sample and 36.4% for the extant specimens, Supplementary Table S1). The sex was established from previous studies to the exception of the extant human sample for which the information was found in the collection record. In 10% of the cases, the information of the sex was lacking and sex determination was established on the secondary sexual characteristics of the skull (see^56,57^). The sex ratio (n females/n total × 100) for each sample is indicated in Supplementary Table S1.

### Methods

To assess the pattern of morphological variation of the MUP specimens, we ran two main analyses (1-MUP fossils morphological affinity and 2-MUP within-group morphological variance) using 57 landmarks and 182 semi-landmarks (i.e. n total = 239, 45 medial and 194 bilateral, Fig. 1, Supplementary Table S2) selected to maximise the description of the morphology of the full cranium. The semi-landmarks were distributed along the cranial sutures and curves joining craniometric points (Supplementary Table S2). The selected landmarks and semi-landmarks aim at capturing as much as possible of the phylogenetic signal from the cranium. Some part of the cranium (i.e. mid-face and upper jaw) have been found to be more influenced by adaptive factors (e.g. diet and climate, see^58^), they nevertheless still correlate with population history^59,60^. All semi-landmarks are technically curve semi-landmarks (two degrees of freedom) and not surface semilandmarks (three degrees of freedom). Missing landmarks were estimated by mirroring and, where mirroring was impossible, by a thin-plate-spline (i.e. TPS) interpolation using the available landmarks^61^. The TPS estimation was based on a global average from the entire sample. This approach was applied to 18 specimens (13 fossils and five extant human individuals) to estimate less than 20% of the landmarks (only three specimens presented more than 5% of missing landmarks, see Supplementary Table S1). Each landmark data set was then superimposed using a Generalized Procrustes Analysis (i.e. GPA, see^47,48^, which removes scale, position and orientation for each specimen in order to focus the analysis on the shape component (i.e. Procrustes shape coordinates) only. To correct for bilateral asymmetry, we used symmetrized landmark configuration of each specimen for subsequent analyses^62^. Outliers from the original dataset were checked for both sets of analyses (see Supplementary Figs. S6 and S8).

#### MUP fossils morphological affinity

The first set of analysis focused on morphological variations of the MUP fossils compared to the fossil samples of EUP and LUP specimens. After alignment of the landmark dataset (i.e. GPA), the Procrustes residuals were used to compute Principal Component Analyses (i.e. PCA) and the first 15 PCs (representing 91.64% of the total variation of the data, Supplementary Table S3) were used to compute between-group PCAs (i.e. bgPCA) in order to reduce the dimensionality of our data before running the bgPCA (i.e. 15 variables for 26 specimens). To visualize the difference in cranial size between the samples, we built boxplots using the centroid size extracted from the results of the GPA (Supplementary Fig. S9). Allometry (i.e. the influence of size on shape) was tested on the first 15 PCs (91.64% of the variation, see Supplementary Fig. S7 and Table S3) considering the sexual attribution of the specimens. We run linear regressions of the shape variables (PC scores) against the size variable (i.e. log(centroid size)). The shape variation was represented for each analysis through a TPS-warped modern human individual (not included in the analyses). To facilitate visualization of shape differences between group means (bgPCA) and between large and small specimens (allometry), we presented a colour map produced by comparing the corresponding surface warps. The colour map is the result of a surface deviation analysis between a reference and a test surface warp. It corresponds to the vector field computed by the difference of the vertex positions of corresponding vertices in both average surface warps. Each vertex is attributed a colour ranging from blue (negative deviation) to red (positive deviation). Mahalanobis, Procrustes (computed from the aligned 3D coordinates) and Euclidean distances (computed from the first 15 PCs) between predetermined groups (i.e. EUP, MUP, LUP and MUPmor, MUPswf, LUPswf, and LUPita) were used to clarify the phenetic relationships within the MUP sample and of the MUP sample with the other Europeans UP fossil samples. Pairwise Mahalanobis, Procrustes and Euclidean distances among all possible pairs of groups (group centroid) were computed and associated p-values were computed by permutation test (10,000 rounds). The use of additional statistical analysis run on the original data (Mahalnobis and Procrustes) as well as cross-validation in the bgPCA, are intended to reduce possible interpretation problems of the bgPCA results (see^63^).

#### MUP within-group morphological variance

The second set of analyses concentrates on the within-group variation of the MUP and LUP specimens. It includes the four extant modern human groups (i.e. AmNat, EuS, OcPap and Inuit) as a comparative sample for the expected variation within modern human groups. The North American and Southern European samples represents human groups, define by their approximate geographical origin, while the Inuit and the Papuan samples represent ethnically defined groups. The EUP sample was excluded from these analyses due to the low number of available specimens (i.e. n = 4). After the Procrustes superimposition of both datasets, we used the Procrustes residuals to compute a PCA (Supplementary Table S7). Allometry did not have a significant impact on shape on the first six PCs (58.00% of the variation, see Supplementary Table S6 and Fig. S10) and the coordinates of the 6 first PCs were used to compute the covariance matrix of each group. Proportionality between covariance matrices of pair of groups are tested (i.e. Maximum likelihood test of proportionality) and the ratios of the generalized variances of the covariance matrices are computed giving an indication of the variation within and between the different samples (i.e. MUP, LUP, AmNat, EuS, OcPap and Inuit, see^64^).

Analyses were performed on the R platform using the Morpho (v 2.1^65^) and Geomorph (v 2.1.2^66^) packages for the 3D geometric morphometric analyses, the Ade4 (v 1.7-4^67^) package for the bgPCA, vcvComp package (v 1.0.1^64^) for the study of the variation within and between groups and ggplot2 (v 3.3.0^68^) to perform graphical representations. Mahalanobis distances were calculated using the MorphoJ software (v 2.0^69^) and surface deviation analysis was performed with Geomagic Studio v.2013.0.1.

## Supplementary Information

Supplementary Information 1.

Supplementary Table S2.

## Data Availability

All data analysed in this study (i.e. the 3D coordinates of the landmarks) are available in the Supplementary Table S2.
