# Supplementary material for: Gravettian cranial morphology and human group affinities during the European Upper Palaeolithic
Source: Sci Rep. 2020 Dec 14;10:21931. doi: 10.1038/s41598-020-78841-x (PMC7736346; doi:10.1038/s41598-020-78841-x)
Supplement: Supplementary file 1 — Supplementary Information 1. [file 41598_2020_78841_MOESM1_ESM.docx]

**Gravettian cranial morphology and human group affinities during the European Upper Palaeolithic**

Aurélien Mounier, Yann Heuzé, Mathilde Samsel, Sergey Vasilyev, Laurent Klaric, Sébastien Villotte

**Supplementary Information**

1. **Fossil sample description**

The selection of fossils included in the analyses was dependent on two criteria. On the one hand, the need for good preservation of the neurocranium and the face. On the other hand, a definite attribution to one of the three Upper Palaeolithic phases distinguished in the article: the Early Upper Palaeolithic (EUP), the Mid-Upper Palaeolithic (MUP) and the Late Upper Palaeolithic (LUP). Most of these fossils (15/26) have been directly C14 dated, and the others come from well-dated and well typochronologically characterized archaeological layers.

The C14 dates presented here are calibrated using IntCal13 and OxCal 4.3^1,2^ and the 95.4% probability ranges are provided.

***EUP fossils***

**Sungir**

The site of Sungir' is located on the eastern edge of the city of Vladimir, approximately 200 km east of Moscow (Russia). It is an open-air site located within loessic deposits on a promontory overlooking the river Klyazma. It has delivered the human remains of at least seven individuals, including two extremely elaborated burials^3^. The lithic assemblage found at the site completely lacks any backed lithics (typical of the Gravettian technocomplex) and Aurignacian index fossils. However, the assemblage does contain distinctive bifacially-worked concave-based triangular points, known as Streletskian points^4,5^. These are found at other Russian sites which date to ~33,000–28,000 ^14^C BP, approximately the same time as Sungir'^6-9^. These sites are typically described as Streletskian, although Sungir' is sometimes also called Sungirian.

Sungir' 1 (Supplementary Fig. S1a) is a single extended burial of an adult mature male^10,11^. Hydroxyproline radiocarbon dates for Sungir' suggest that the site, including this burial, date to ~30,000–29,000 ^14^C BP (~34,500–32,800 cal BP^12,13^). Sungir' 1 skull does not display any obvious traumatic injuries or deformation, but the midline of its frontal bone “has a pronounced external curvature along the midline, located in the vicinity of metopion” ^11^. This does not place Sungir' 1 as an outlier in nasion-bregma arc and chord comparisons^11^.

**Kostënki 14**

The site of Kostënki 14 (Markina Gora) contains a deep stratified open-air sequence where a series of archaeological layers dating from the very beginning of the Upper Palaeolithic to the late Mid Upper Palaeolithic have been found^14-18^. The site is one of 21 Upper Palaeolithic sites located in the village of Kostënki, on the western bank of the Don river approximately 40 km south of Voronezh.

The crouched human burial known as Kostënki 14 (Supplementary Fig S1b), an adult male^19^, has been directly dated using the single amino acid (hydroxyproline) method to 33,900 ± 550 ^14^C BP (39,700–36,800 cal BP; OxA-X-2395-15; corrected date^12^). This is very similar to dates for the Kostënki 14/LVA Early Aurignacian assemblage^14,17,20^.

**Mladeč**

The finds from Mladeč derive from an extensive karstic system in the Třesín hill located close to the Morava river in Moravia, Czechia. The human remains and archaeological finds from the cave system seem to have entered the system from the exterior via fissures^21^.

The Mladeč 1 adult female cranium^22^ (Supplementary Fig S1c) is the most complete cranium from the site. Numerous other human remains and archaeological finds including Mladeč-type massive-based bone points were also found inside this cavity^21,22^. A tooth from Mladeč 1 was dated to 31,190 +400/-390 ^14^C BP (36,000–34,400 cal BP; VERA-3073), in line with dates for other human teeth from Mladeč^23^. The dating of the human remains and the archaeological finds from the cave, especially the massive-based bone points, support an attribution to the late Aurignacian period. The Mladeč 1 cranium is free from external pathological lesions or abnormalities^22^.

**Peştera Muierii**

The Peştera Muierii is a complex karstic system located at Baia de Fier, on the southern edge of the Southern Carpathian Mountains in Romania. Excavations took place in the cave system between 1951 and 1955. Six human bones were discovered in the cave, four of them (cranium, mandible, scapula and tibia) being interpreted as belonging to a single individual, the mature female Muierii 1^24,25^ (Supplementary Fig. S1d). A combined sample from the tibia and scapula was radiocarbon dated to 30,150 ± 700 ^14^C BP (35,700–32,900 cal BP; LuA-5228) while a sample from the cranium was later dated to 29,930 ± 170 ^14^C BP (34,400–33,700 cal BP; OxA-15529)^25,26^. The dates for the remains suggest that they date to the late Aurignacian period.


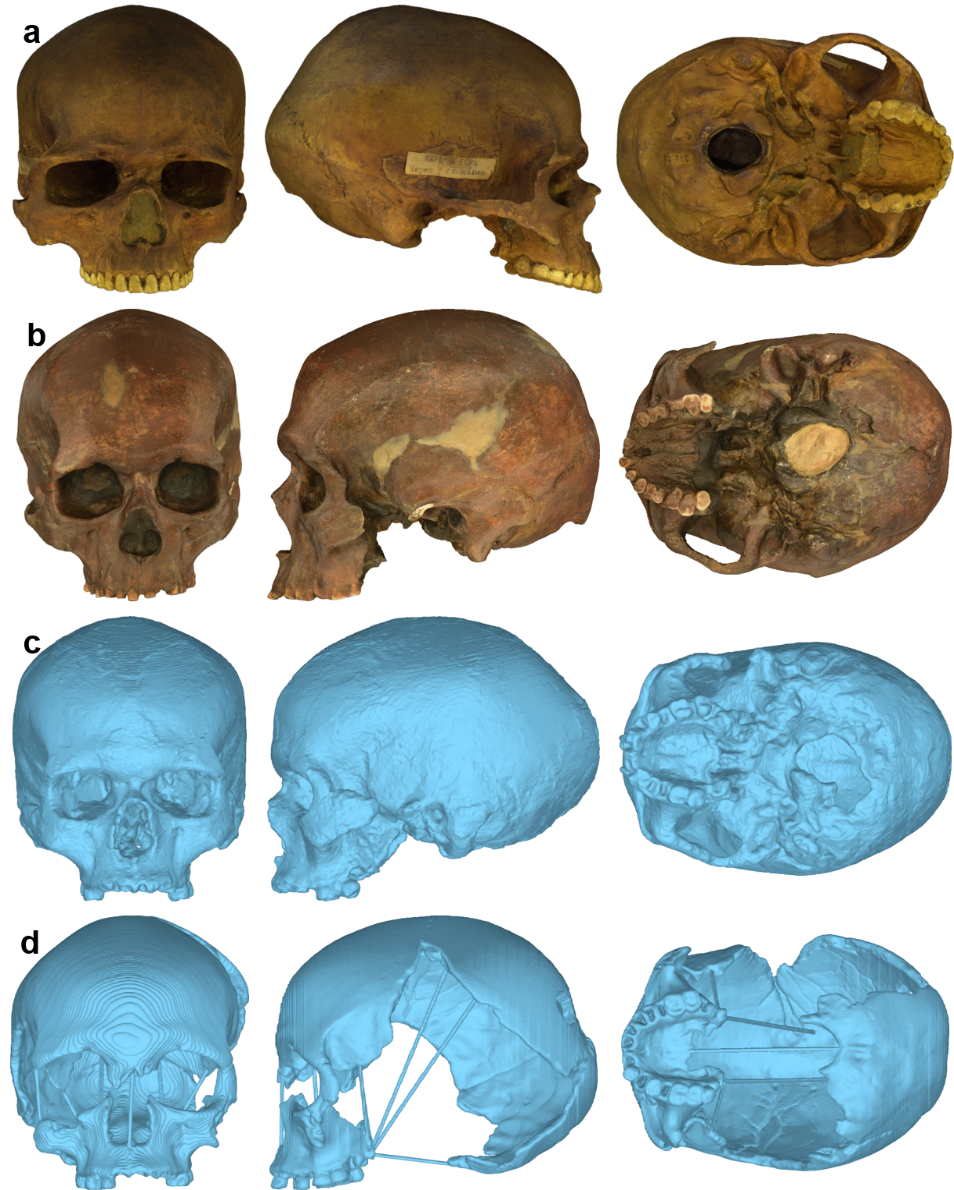


**Figure S1.** *Norma frontalis, norma lateralis* and *norma basalis* of a) Kostënki 14, b) Sungir’ 1, c) Mladeč 1 and d) Muierii 1. The specimens are not to scale.

***MUP fossils from Moravia (Czechia)***

**Dolní Věstonice-Pavlov**

The Dolní Věstonice and Pavlov sites (Dolní Věstonice I, II, IIa, III; Pavlov I–VI) are located on the northern edge of the Pavlov hills overlooking the Dyje river valley in southern Moravia, Czechia. The majority of activity at the sites is attributed to the Evolved Pavlovian phase, dating to ~27,000–25,000 ^14^C BP^27,28^.

The Dolní Věstonice 3 individual is an adult female (Supplementary Fig. S2a). There are no direct dates for the burial but dates for the cultural layer in the vicinity of the burial fall in the range ~27,000–25,000 ^14^C BP, like the DV 13–15 and DV 16 burials^29,30^.

The remains of the three individuals, late teenagers / young adults males, known as Dolní Věstonice 13–15 were discovered within a single burial pit^28^. These three individuals were recently directly dated to ~31,000 cal BP^30^:

- DV13 (Supplementary Fig. S2b): 27,040 ± 100 BP (31,250–30,880 cal BP; Aix-12027)

- DV14 (Supplementary Fig. S2c): 26,760 ± 100 BP (31,120–30,730 cal BP; Aix-12028)

- DV15 (Supplementary Fig. S2d): 26,680 ± 70 BP (31,110–30,720 cal BP; Aix-12029)

The Dolní Věstonice 16 individual is an adult male^28^ (Supplementary Fig. S2e) directly dated to 27,220 ± 110 BP (31,350–30,970 cal BP; Aix-12030^30^).

DV3 cranium displays an important degree of facial and neurocranial asymmetry which is responsible for abnormal gross morphology^28^, which places it sometimes as an outlier in our analyses (see text). Despite many appendicular abnormalities, DV15 does not exhibit any cranial clear pathological morphologies^28^. DV16 facial remains display minor deformation related to a growth abnormality or a traumatic injury^28^.

**Předmostí I**

The site of Předmostí I is located on the outskirts of the town of Přerov in Moravia, Czechia, overlooking the Bečva river valley. The remains of ~20 individuals were discovered in a single small area^31^. The taphonomy of the burial area suggest that it is not a single mass grave created in one event, but rather the location of repeated burials over some period of time, in some cases disturbing earlier burials^31^. There is a lower layer at the site containing a Pavlovian assemblage and an upper layer containing a Kostënki-Willendorfian assemblage. The burials are believed to be associated with the Pavlovian assemblage. There are several dates for the Pavlovian layer at the site that fall in the range 26,000–25,000 ^14^C BP^31^ (~30,000–29,000 cal BP).

The human skeletal material from Předmostí was destroyed at the end of World War II but casts of the Předmostí 3 (Supplementary Fig. S2f) and 4 (Supplementary Fig. S2g) crania, as well as photographs, are available for study. Předmostí 3 is an adult male^32^. Doubts remain regarding the association of skull Předmostí 4 with post-cranial skeleton Předmostí 4^32^. Nevertheless, both old^33^ and recent^32^ analyses provide a female assessment for this skull.


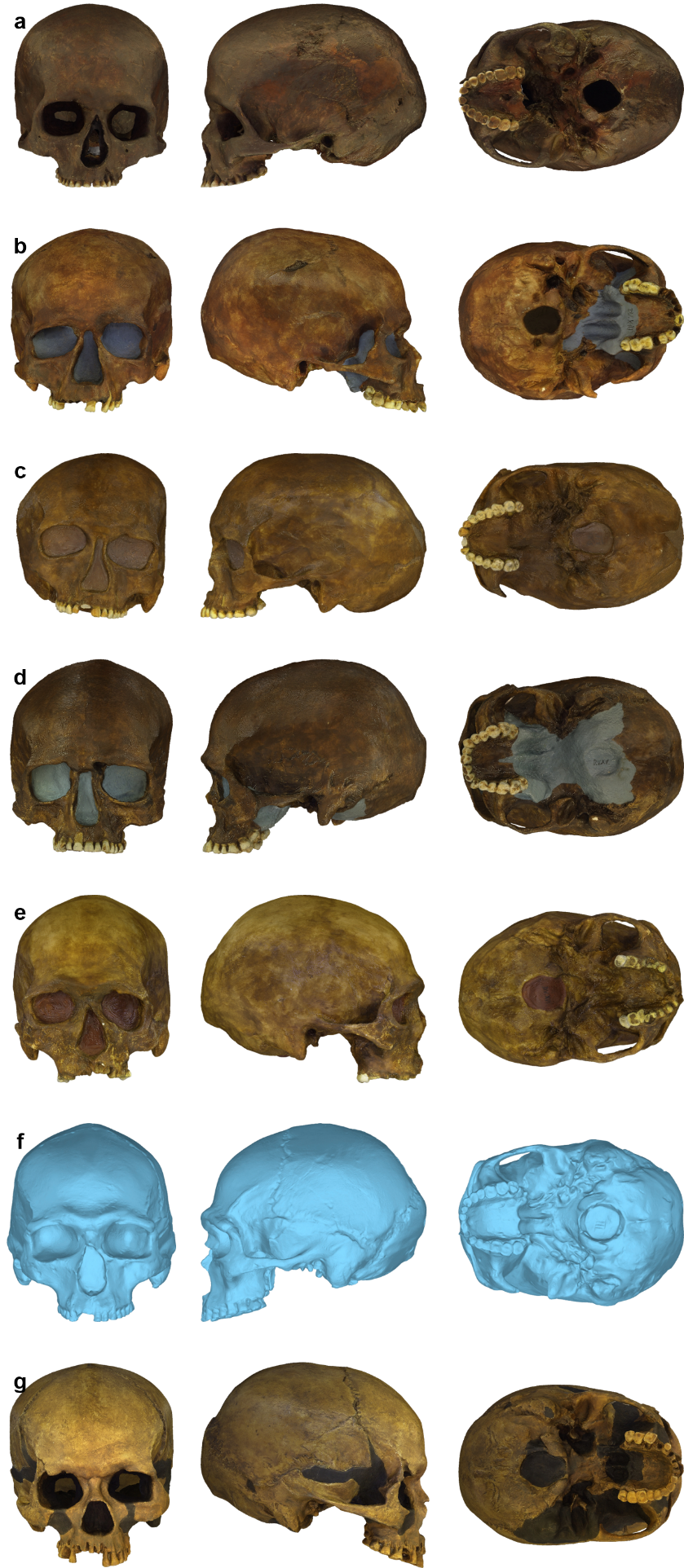


**Figure S2.** *Norma frontalis, norma lateralis* and *norma basalis* of a) Dolní Věstonice 3, b) Dolní Věstonice 13, c) Dolní Věstonice 14, d) Dolní Věstonice 15, e) Dolní Věstonice 16, f) Předmostí 3, and g) Předmostí 4. The specimens are not to scale.

***MUP fossils from SW France***

**Cro-Magnon**

The site of Cro-Magnon is located in the village of Les Eyzies de-Tayac-Sireuil (Dordogne, France) close to the Vézère river. At the end March 1868, human remains morphologically similar to recent humans and associated with prehistoric artifacts and extinct fauna were discovered by workers at that site^34^. This discovery quickly led to excavations in or around the shelter and, in less than 50 years, it was emptied^35^. The site delivered the human remains of at least height individuals, four adults and four young children^36-38^. These human remains were for long time considered as dating from the Early Aurignacian but it has been demonstrated that they are associated to the Early phase of the Gravettian, based on stratigraphic comparisons with Abri Pataud Layer 5 (the two sites are located 300 meters apart) and a direct ^14^C date of 27,680 ± 270 BP (32,700–31,300 cal BP; Beta-157439) on an adornment associated to the burial^35,39^.

Cro-Magnon 1 skull (Supplementary Fig. S3a) can be securely associated to individual Alpha, an old male^38^. This skull displays a frontal erosive lesion^37^ and one of the 239 landmaks which should have been positioned inside the lesion was considered missing and estimated. Cro magnon 2 skull (Supplementary Fig. S3b) most likely belongs to a female based on cranial sexually dimorphic size and shape variables, though discriminant function analyses do not ascertain this attribution ^40^. This skull may be associated to individual Beta, an aged adult female^38^.

**Cussac**

The decorated Grotte de Cussac (Le Buisson-de-Cadouin, Dordogne, France) was discovered in 2000. The cave revealed associated parietal art, prehistoric surfaces, and human remains belonging to at least 6 individuals^41,42^. It was designated a national heritage site and remains closed to visitors. All research observations are non-invasive and restricted to a single path through the karstic tube in order to protect all traces of human and faunal activity. The cave has nonetheless been studied since 2010. The human activity in Cussac is attributed to the Gravettian, based on the art, which displays features typical of the Middle Gravettian style and similarities with the parietal art of Quercy and the Pyrenees and on two ^14^C dates: one on charcoal from the cave floor (25,150 ± 210 BP; 29,704–28,714 cal BP; GifA-13150) and one on a human rib fragment from Locus 1 Depression 2 (25,120 ± 120 BP; 29,500–28,800 cal BP; Beta-156643^42^).

Only one skull has been discovered so far in the cave^41^: it belongs to Cussac L2A (Supplementary Fig. S3c), an adult male represented by a virtually complete skeleton^40,43^. L2A displays an unusual cranial morphology of unknown origin, although it cannot be considered a clear outlier^40^.

**Abri Pataud**

The site of Abri Pataud is located in the commune of Les Eyzies de-Tayac-Sireuil (Dordogne, France) above the Vézère river. This rock shelter infill was excavated by Movius between 1953 and 1964 and attains a thickness of 9 m. Fourteen archaeological levels separated by sterile scree were identified, with a sequence extending from the early Aurignacian to the Solutrean^44^.

Level 2 delivered more than 400 human remains, belonging to at least 6 individuals^45-47^. Level 2 is attributed to the final Gravettian based on the typotechncological analysis of the lithic material and it is dated to 28,000–26,000 cal BP^44,46^. The skull under study belongs to Abri Pataud 1 (Supplementary Fig. S3d), a young adult female^45-47^.

**
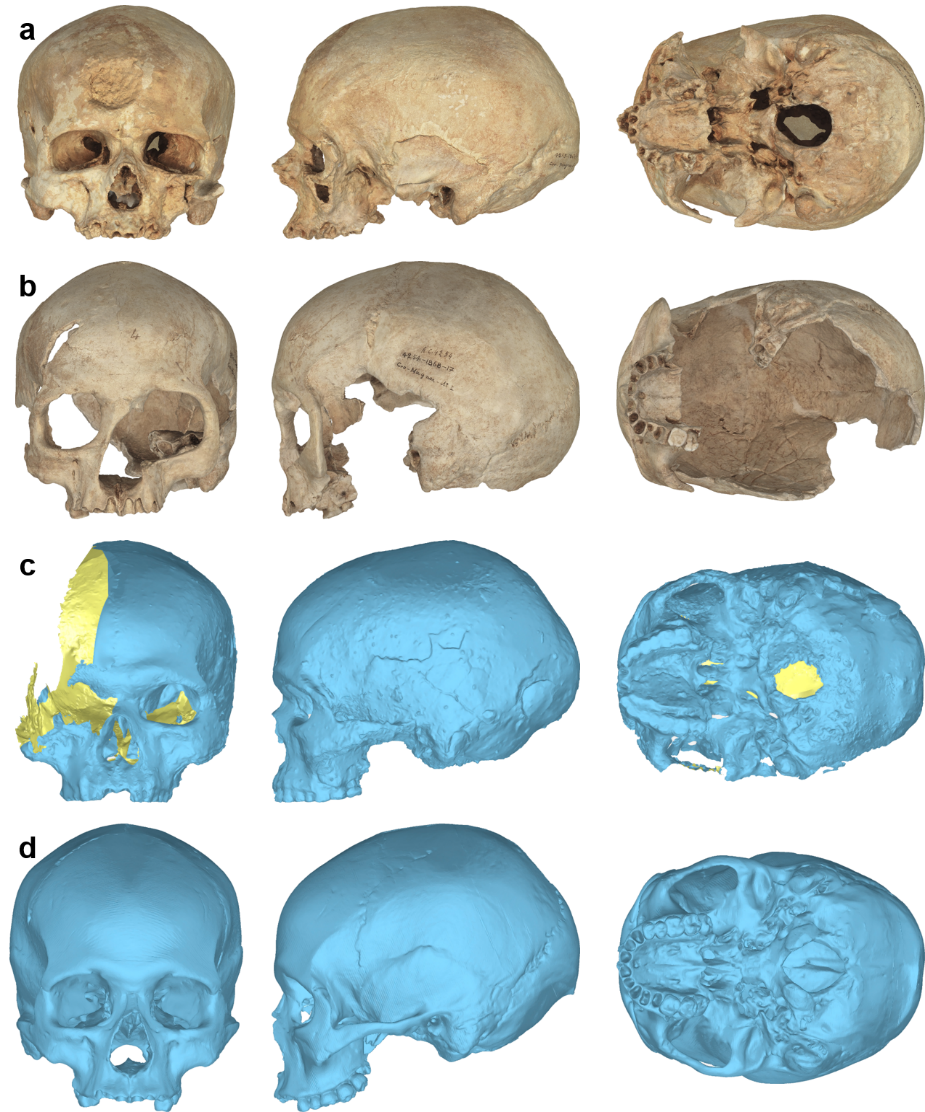
**

**Figure S3.** *Norma frontalis, norma lateralis* and *norma basalis* of a) Cro-Magnon 1, b) Cro-Magnon 2, c) Cussac L2A, and d) Abri Pataud 1. The specimens are not to scale.

***LUP fossils from South West France***

**Chancelade**

The Raymonden cave, at Chancelade (Dordogne, France), close to Périgueux, has been excavated since 1887. The site has mainly delivered artefacts associated to several subperiods of the Magdalenian^48^. In 1888, Hardy discovered the partial skeleton of an adult individual covered with ochre ^49^. This individual (Supplementary Fig. S4a) is usually considered as male, based on cranial morphological analyses^48,50^, but this sex assessment appears problematic^10^. A direct C14 date obtained from the skeleton^51^ provided a date of 15,035 ± 70 (18,031–18,480 cal BP; OxA-29885). This date allows to associate Chancelade skeleton to the early phase of the Middle Magdalenian^51,52^. Chancelade cranium displays a minor depression in right temporoparietal region, which may be a healed traumatic injury^53^.

**Lafaye**

The Lafaye rochselter is located at Bruniquel (Tarn-et-Garonne, France), in a limestone cliff above the Aveyron river. The skeletal remains of an adult (Lafaye 24, Supplementary Fig. S4b) and an infant (Lafaye 25) were discovered in 1865 and considered as dating from prehistoric times since their discovery, but were formally associated to the Magdalenian only a century later^54^. This attribution was confirmed by a direct dating of the Lafaye 24: 15,290 ± 150 BP (GifA95047) 18,856–18,189 cal BP^55^, which places these fossils at the early phase of the Middle Magdalenian^51,52^. Lafaye 24 is a young or mature female^10^. The skull exhibits minor depressions on the frontal and parietal bones that may be related to heal traumatic injuries (Villotte pers. obs.).

**Saint-Germain-la-Rivière**

The Saint-Germain-la-Rivière site is located in Pillé-Bourse (Gironde, France), on the right bank of the Dordogne. It has been excavated several times since 1929 and delivered four main layers, one attributed to the early Magdalenian, two to the middle Magdalenian, and one to the late Magdalenian/Azilian^56^. A burial of a one individual associated with a rich set of grave goods was discovered in 1934 by Blanchard directly below the ceiling of the shelter of the upper terrace^57^. A direct AMS ^14^C date^55^ of this skeleton (Saint-Germain-la-Rivière 4) provided a date of 19.500—18.600 cal BP (15 780 ± 200 BP; GifA-95456) for this individual, allowing its association to the early phase of the Middle Magdalenian^51,52^. Saint-Germain-la-Rivière 4 (Supplementary Fig. S4c) is a young adult female^10,58^.

**Cap blanc**

The Cap Blanc shelter is located ~8 km from Les Eyzies-de-Tayac-Sireuil (Dordogne, France). It opens into a south-facing conical limestone outcrop on the right bank of the Grande Beune valley. This shelter is famous for Palaeolithic sculpted frieze associated to the Magdalenian culture. The site was discovered in 1909 and almost emptied very quickly^58^. A burial at the base of the archaeological deposit was discovered in 1911^59^. Several Palaeolithic chrono-cultural entities dated to the LGM and the pre LGM, have been identified at Cap Blanc, but the main occupation is associated to the Middle Magdalenian^60,61^. The burial is undoubtedly a pre-LGM Palaeolithic burial^60,61^ and, according to Barshay-Szmidt, et al.^51^, very likely associated to the group of burials from South-West France associated the early phase of the Middle Magdalenian (19,000-17,500 cal BP, see^52^). The skeleton (Supplementary Fig. S4d) is considered belonging to a late adolescent / young adult female (Bonin, 1935).

**Le placard**

Known since the middle of the 19^th^ century, the site of Le Placard (Vilhonneur, Charente, France) has delivered archaeological material associated to the Middle Palaeolithic, Upper Palaeolithic, Neolithic and historical times^62^. In 1881, a complete cranium (Supplementary Fig. S4e) and its associated mandible was discovered at Le Placard and attributed to the “Age of the Reindeer”^63^ which was the terminology for the Magdalenian culture at the end of the 19^th^ century. The recent reanalysis of near 200 human remains from Le Placard^62^ associated most of them to the Badegoulian chronoculture (~22,000 cal BP). However, a small set of bones were not associated to the period, including the skull and the mandible which has been directly dated to 15,480 ± 70 BP (18,890-18,590 cal. BP; Lyon-6498(GrA)) and may thus be associated to the early phase of the Middle Magdalenian. The skull is considered belonging to a female individual^63^.

**
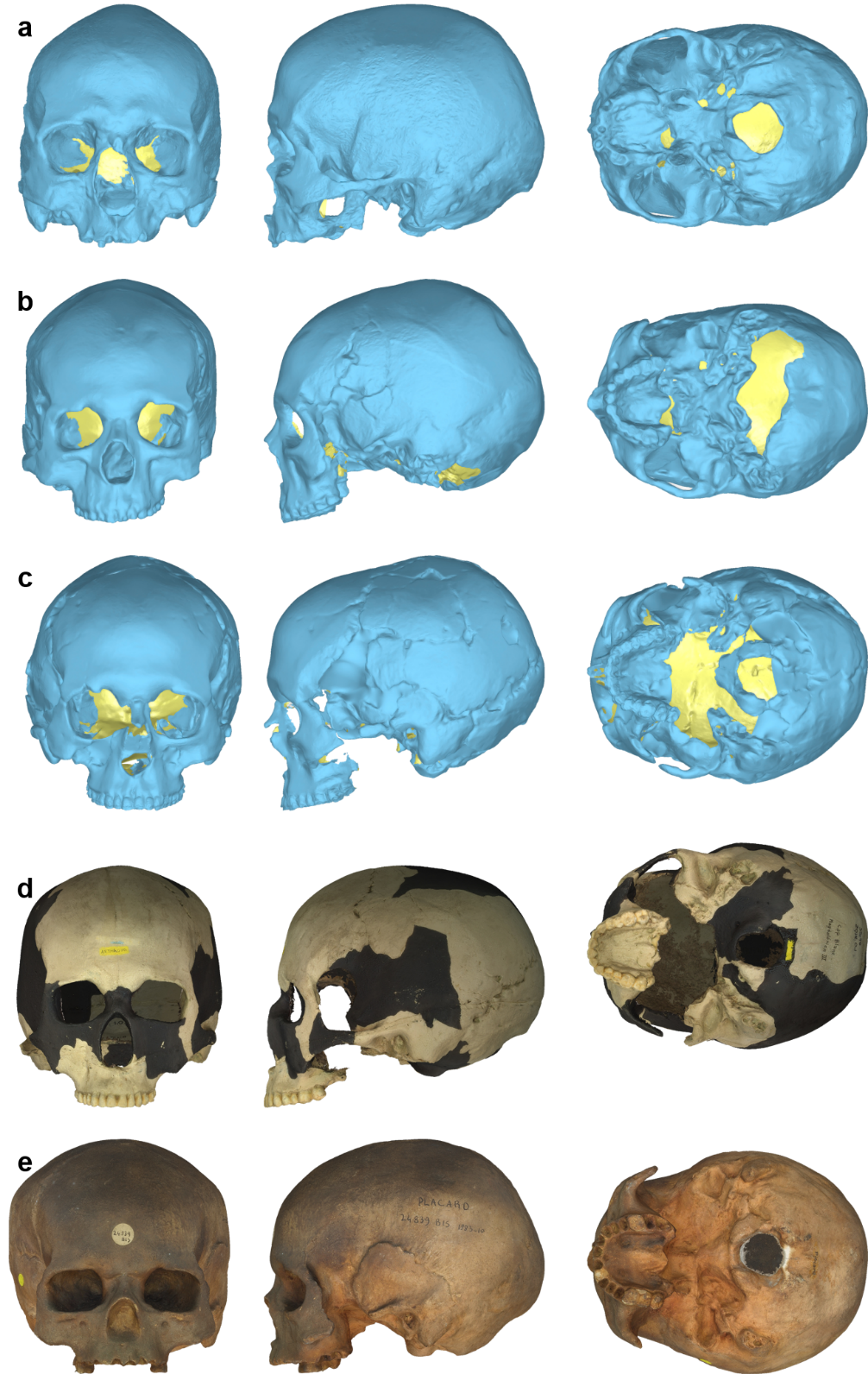
**

**Figure S4.** *Norma frontalis, norma lateralis* and *norma basalis* of a) Chancelade 1, b) Lafaye 24, c) Saint-Germain La Rivière 4, d) Cap Blanc and e) Le Placard 142. The specimens are not to scale.

***LUP fossils from Italy***

**Arene Candide**

Arene Candide is a large cave system accessed 90 m above current sea level on the slopes of Monte Caprazoppa (Finale Ligure, Italy). The site is known since the latter half of the 19th century and excavations have taken place regularly since then^64^. Several Late Upper Palaeolithic burials and secondary deposits of human bones were unearthed at Arene Candide (mostly during the 1940-1942 excavation), with a minimum number of individuals of 20^64^. This necropolis culturally attributed to the Final Epigravettian^65^. Direct ^14^C AMS dates obtained from human skeletons are distributed in two distinct groups that indicate two phases of funerary utilisation of the cave^66^.

Based on archeological and archeothanotological evidences^64^, Arene Candide 2, 3, and 4 (respectively, Supplementary Fig. S5a, b, and c) are contemporaneous to one another, and associated to the second phase of the Epigravettian occupation of the Cave (12,030-11,180 cal BP). Arene Candide 2 failed to yield sufficient collagen for AMS ^14^C dating, and Arene Candide 4 has not been directly dated^64,66^. Arene Candide 3 has been directly dated to 10,065± 55 BP (11,940–11,330 cal BP; OxA-10998)^66^. These individuals are three adult males^10,67^. Arene Candide 2 and 3 are both affected by a systemic condition, but no cranial abnormalities were identified for Arene Candide 3 and only a minor frontal bossing was described for Arene Candide 2^64^.

**Villabruna**

The rock shelters named “Ripari Villabruna” are located in Val Cismon (Sovramonte, Belluno, Italy). The archaeological deposit suggests various short-term anthropogenic events, starting during the Late Epigravettian. In 1988, Broglio discovered a burial of a young adult male at the base of the archaeological deposit^68,69^. The direct AMS date of the skeletal remains of Villabruna 1 (12,140±70 BP; KIA-27004; calibrated: 14,160–13,820 BP, Supplementary Fig. S5d) confirmed the attribution to the Late Epigravettian^68^.

**San Teodoro**

Grotta di San Teodoro is situated between the cities of Palermo and Messina, in Sicily. Excavations at the cave started in 1859. Human remains of several individuals, including a burial with a well-preserved skeleton (San Teodoro 1) and two crania (San Teodoro 2 and 7), were discovered in the same stratigraphic horizon in 1937^70^. These human remains are considered as contemporaneous as they were discovered under a five-centimetre-thick deposit of red ochre, in a layer attributed to the late Epigravettian culture^70-72^.

San Teodoro 1 is a young adult individual^10,73^ (Supplementary Fig. S5e). Three independent analyses of the San Teodoro 1 pelvis provided a female assessment^10,73,74^, while a fourth one provided a male diagnosis^75^. We thus considered this individual as “female?”. San Teodoro 1 was directly dated to 12,580 ± 130 BP (15,232–14,126 cal BP; ETH-34451)^70^. San Teodoro 2, a well-preserved skull (Supplementary Fig. S5f), is considered to belong to an adult male based on cranial morphology^72^.


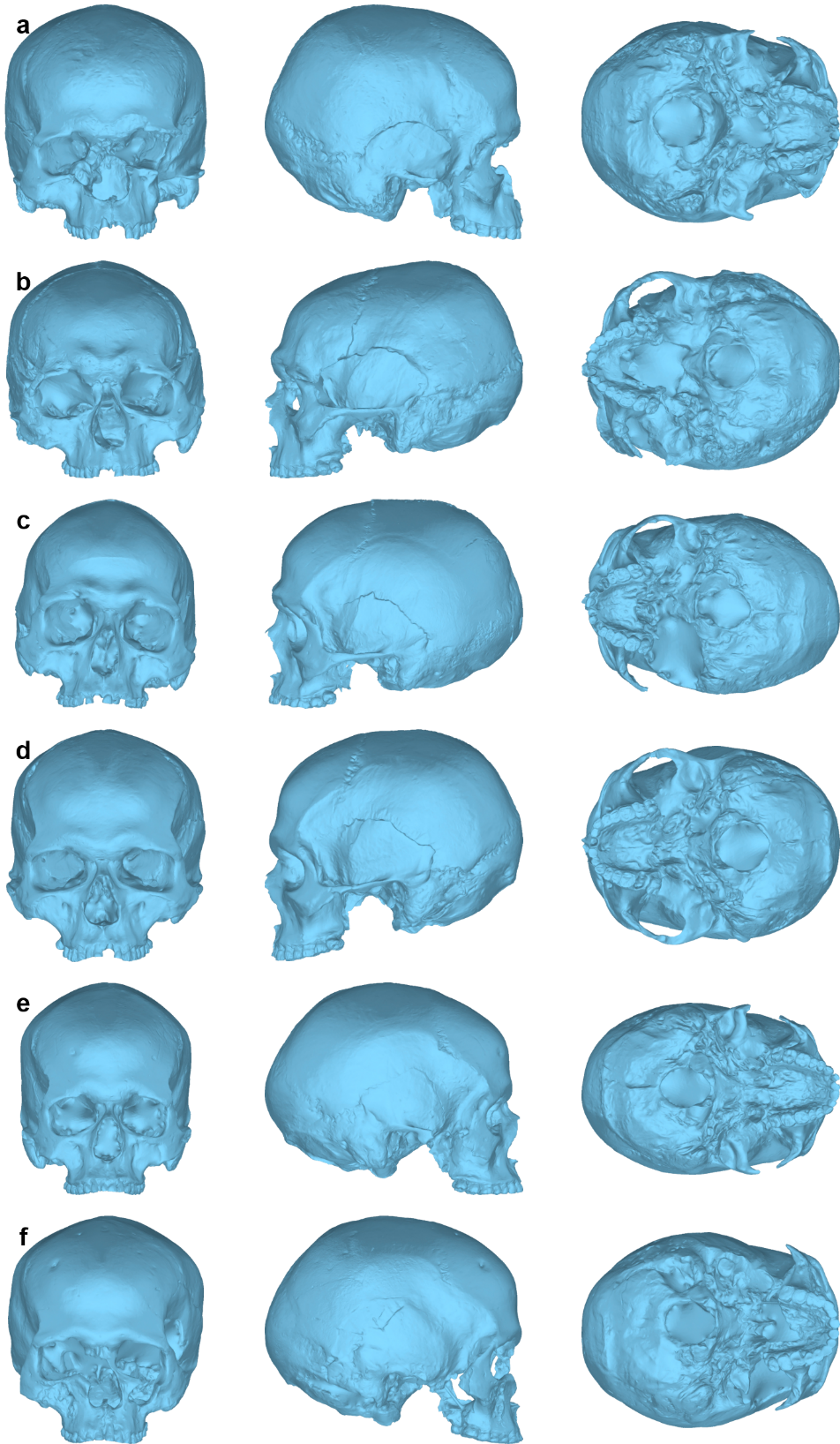


**Figure S5.** *Norma frontalis, norma lateralis* and *norma basalis* of a) Arene Candide 2, b) Arene Candide 3, c) Arene Candide 4 d) Villabruna 1, e) San Teodoro 1, and f) San Teodoro 2. The specimens are not to scale.

1. **Full sample description**

**Table S1.** Description of the full crania sample used in the study. Specimens in bold: data from the original specimens.

| **Specimens** | **Chronology** | **References** | **Sex** | **Site / Country** | **Technocomplex** | **3D^µ^** | **Inst.*** | **Labels** | **Group Labels** | **n F / n Sp** | **% missing landmarks** |
| --- | --- | --- | --- | --- | --- | --- | --- | --- | --- | --- | --- |
| ***Upper Palaeolithic populations*** | |  |  |  |  |  |  |  |  | 0.42 |  |
|  |  |  | ***EUP*** |  |  |  |  |  |  | 0.50 |  |
| Kostënki 14 | 39,700–36,800 cal BP | ^12^ | M | Kostënki-Borshchyovo, Russia | Aurignacien | PH | Brno | Ko14 | EUP |  | 0.0 |
| **Sungir’ 1** | 34,500–32,800 cal BP | ^12,13^ | M | Sungir’, Russia | Sungirian | PH | IEA | Sun | EUP |  | 0.4 |
| **Mladeč 1** | 36,000–34,400 cal BP | ^23^ | F | Mladeč, Czech Republic | Aurignacien | CT | NM | Ml1 | EUP |  | 0.0 |
| **Muierii 1** | 34,400–33,700 cal BP | ^25^ | F | Peştera Muierii, Romania | Aurignacien | CT | ACB | Mui | EUP |  | 19.2 |
|  |  |  | ***MUP*** |  |  |  |  |  |  | 0.42 |  |
| **Cro-Magnon 1** | 32,700–31,300 cal BP | ^35,39^ | M | Cro-Magnon, France | Gravettian | PH | MH | CM1 | MUPswf |  | 0.4 |
| **Cro-Magnon 2** | 32,700–31,300 cal BP | ^35,39^ | F? | Cro-Magnon, France | Gravettian | PH | MH | CM2 | MUPswf |  | 9.2 |
| **Cussac L2A** | 29,500–28,800 cal BP | ^42^ | M | Cussa, France | Gravettian | PH | DRACNA | Cus | MUPswf |  | 0.4 |
| **Abri Pataud 1** | 28,000–26,000 cal BP | ^44^ | F | Abri-Pataud, France | Gravettian | CT | MH | AP1 | MUPswf | 0.50 | 0.0 |
| **Dolní Věstonice 3** | 31,000–30,000 cal BP | ^29^ | F | Dolní Věstonice I, Czech Republic | Gravettian | PH | Brno | DV3 | MUPmor |  | 0.0 |
| Dolní Věstonice 13 | 31,250–30,880 cal BP | ^30^ | M | Dolní Věstonice II, Czech Republic | Gravettian | PH | Brno | DV13 | MUPmor |  | 0.0 |
| Dolní Věstonice 14 | 31,120–30,730 cal BP | ^30^ | M | Dolní Věstonice II, Czech Republic | Gravettian | PH | Brno | DV14 | MUPmor |  | 0.0 |
| Dolní Věstonice 15 | 31,110–30,720 cal BP | ^30^ | M | Dolní Věstonice II, Czech Republic | Gravettian | PH | Brno | DV15 | MUPmor |  | 0.4 |
| Dolní Věstonice 16 | 31,350–30,970 cal BP | ^30^ | M | Dolní Věstonice II, Czech Republic | Gravettian | PH | Brno | DV16 | MUPmor |  | 0.0 |
| Předmostí 3 | 30,000–29,000 cal BP | ^31^ | M | Předmostí, Czech Republic | Gravettian | OP | DL | Pr3 | MUPmor |  | 0.0 |
| Předmostí 4 | 30,000–29,000 cal BP | ^31^ | F | Předmostí, Czech Republic | Gravettian | PH | Brno | Pr4 | MUPmor | 0.29 | 0.0 |
|  |  |  | ***LUP*** |  |  |  |  |  |  | 0.36 |  |
| Cap Blanc | 19,000–17,500 cal BP | ^51^ | F | Abri du Cap Blanc, France | Magdalenian | PH | MH | CB | LUPswf |  | 1.3 |
| **Chancelade 1** | 18,031–18,480 cal BP | ^51^ | M? | Chancelade, France | Magdalenian | OP | MAAP | Cha | LUPswf |  | 0.0 |
| **Lafaye 24** | 18,856–18,189 cal BP | ^55^ | F | Abri Lafaye, France | Magdalenian | OP | MHN | Laf | LUPswf |  | 0.8 |
| Le Placard 142 | 18,890–18,590 Cal BP | ^62^ | F? | Grotte du Placard, France | Magdalenian | PH | MH | LP | LUPswf |  | 0.8 |
| **Saint-Germain La Rivière 4** | 19,500–18,600 cal BP | ^55^ | F | Saint-Germain La Rivière, France | Magdalenian | OP | MNP | SGLR | LUPswf | 0.80 | 2.1 |
| **Arene Candide 2** | 11,940–11,330 cal BP | ^66^ | M | Caverna delle Arene Candide, Italy | Epigravettian | OP | SAG | AC2 | LUPita |  | 10.5 |
| **Arene Candide 3** | 11,940–11,330 cal BP | ^66^ | M | Caverna delle Arene Candide, Italy | Epigravettian | OP | SAG | AC3 | LUPita |  | 0.3 |
| **Arene Candide 4** | 11,940–11,330 cal BP | ^66^ | M | Caverna delle Arene Candide, Italy | Epigravettian | OP | SAG | AC4 | LUPita |  | 0.0 |
| **San Teodoro 1** | 15,232–14,126 cal BP | ^70^ | F? | Grotta di San Teodoro, Italy | Epigravettian | OP | MPG | ST1 | LUPita |  | 0.0 |
| **San Teodoro 2** | 15,232–14,126 cal BP | ^70^ | M | Grotta di San Teodoro, Italy | Epigravettian | OP | MFP | ST2 | LUPita |  | 0.8 |
| **Villabruna 1** | 14,160-13,820 Cal BP | ^68^ | M | Riparo di Villabruna, Italy | Epigravettian | OP | SAV | Vil | LUPita | 0.17 | 0.0 |
|  |  |  |  |  |  |  |  |  |  |  |  |
| **Specimens** | **Chronology** | **References** | **Sex** | **Country** | **Group** | **3D^µ^** | **Inst.*** | **Labels** | **Group Labels** | **n F / n Sp** | **% missing landmarks** |
| ***Extant populations*** | |  |  |  |  |  |  |  |  | 0.36 |  |
| ***Ethnically defined populations*** | |  |  |  |  |  |  |  |  |  |  |
|  |  |  | **Papua** |  |  |  |  |  |  |  |  |
| **MEL076** | 19-20^th^ c. |  | F | Papua New Guinea | Papuan | PH | DL |  | OcPap |  | 0.0 |
| **MEL084** | 19-20^th^ c. |  | M | Papua New Guinea | Papuan | PH | DL |  | OcPap |  | 0.0 |
| **MEL085** | 19-20^th^ c. |  | M | Papua New Guinea | Papuan | PH | DL |  | OcPap |  | 0.0 |
| **MEL104** | 19-20^th^ c. |  | F | Papua New Guinea | Papuan | CT | DL |  | OcPap |  | 0.0 |
| **MEL130** | 19-20^th^ c. |  | F | Papua New Guinea | Papuan | PH | DL |  | OcPap |  | 0.0 |
| **MEL153** | 19-20^th^ c. |  | F | Papua New Guinea | Papuan | CT | DL |  | OcPap |  | 0.0 |
| **MEL154** | 19-20^th^ c. |  | F | Papua New Guinea | Papuan | CT | DL |  | OcPap |  | 0.0 |
| **MEL197** | 19-20^th^ c. |  | M | Papua New Guinea | Papuan | CT | DL |  | OcPap |  | 0.0 |
| **MEL210** | 19-20^th^ c. |  | F | Papua New Guinea | Papuan | PH | DL |  | OcPap |  | 0.0 |
| **MEL214** | 19-20^th^ c. |  | M | Papua New Guinea | Papuan | PH | DL |  | OcPap |  | 0.8 |
| **MEL217** | 19-20^th^ c. |  | M | Papua New Guinea | Papuan | PH | DL |  | OcPap | 0.54 | 0.0 |
|  |  |  | **Inuit - Greenland** |  |  |  |  |  |  |  |  |
| **NA123** | 19-20^th^ c. |  | M | Denmark (Greenland) | Inuit | CT | DL |  | Inuit |  | 0.0 |
| **NA124** | 19-20^th^ c. |  | F | Denmark (Greenland) | Inuit | CT | DL |  | Inuit |  | 0.0 |
| **NA133** | 19-20^th^ c. |  | F | Denmark (Greenland) | Inuit | CT | DL |  | Inuit |  | 0.0 |
| **NA134** | 19-20^th^ c. |  | M | Denmark (Greenland) | Inuit | CT | DL |  | Inuit |  | 0.0 |
| **NA136** | 19-20^th^ c. |  | M | Denmark (Greenland) | Inuit | CT | DL |  | Inuit |  | 0.0 |
| **NA137** | 19-20^th^ c. |  | M | Denmark (Greenland) | Inuit | CT | DL |  | Inuit |  | 0.0 |
| **NA138** | 19-20^th^ c. |  | M | Denmark (Greenland) | Inuit | CT | DL |  | Inuit |  | 0.0 |
| **NA140** | 19-20^th^ c. |  | M | Denmark (Greenland) | Inuit | CT | DL |  | Inuit |  | 0.0 |
| **NA144** | 19-20^th^ c. |  | F | Denmark (Greenland) | Inuit | CT | DL |  | Inuit |  | 0.8 |
| **NA151** | 19-20^th^ c. |  | F | Denmark (Greenland) | Inuit | CT | DL |  | Inuit |  | 0.0 |
| **NA173** | 19-20^th^ c. |  | F | Denmark (Greenland) | Inuit | CT | DL |  | Inuit | 0.45 | 0.0 |
| ***Geographic populations*** | |  |  |  |  |  |  |  |  |  |  |
|  |  |  | **Native - North America** |  |  |  |  |  |  |  |  |
| **NA23** | 19-20^th^ c. |  | F | USA (New Mexico) | Zuni | CT | DL |  | AmNat |  | 0.4 |
| **NA61** | 19-20^th^ c. |  | M | USA (New Mexico) | Zuni | CT | DL |  | AmNat |  | 0.0 |
| **NA68** | 19-20^th^ c. |  | F | USA (Midwest) | Sioux | CT | DL |  | AmNat |  | 0.0 |
| **NA71** | 19-20^th^ c. |  | M | USA (New Mexico) | Apache | CT | DL |  | AmNat |  | 3.8 |
| **NA72** | 19-20^th^ c. |  | M | USA (Midwest) | Sioux | CT | DL |  | AmNat |  | 0.0 |
| **NA74** | 19-20^th^ c. |  | M | Canada (Ontario) | Iroquois | CT | DL |  | AmNat |  | 0.0 |
| **NA81** | 19-20^th^ c. |  | M | Canada (Ontario) | Huron | CT | DL |  | AmNat |  | 0.0 |
| **NA82** | 19-20^th^ c. |  | M | Canada (Ontario) | Huron | CT | DL |  | AmNat |  | 0.0 |
| **NA83** | 19-20^th^ c. |  | M | Canada (Ontario) | Huron | CT | DL |  | AmNat |  | 0.0 |
| **NA92** | 19-20^th^ c. |  | M | Canada (British Columbia) | Squamish | CT | DL |  | AmNat |  | 0.0 |
| **NA95** | 19-20^th^ c. |  | M | Canada (British Columbia) | Squamish | CT | DL |  | AmNat | 0.18 | 0.0 |
|  |  |  | **South Europe** |  |  |  |  |  |  |  |  |
| **Eu-1036** | 19-20^th^ c. |  | F | France | French | CT | DL |  | EuS |  | 0.0 |
| **Eu-1042** | 19-20^th^ c. |  | M | France | French | CT | DL |  | EuS |  | 0.0 |
| **Eu-1051** | 19-20^th^ c. |  | M | France | French | CT | DL |  | EuS |  | 0.0 |
| **Eu-1067** | 19-20^th^ c. |  | M | France | French | CT | DL |  | EuS |  | 0.0 |
| **Eu-1114** | 19-20^th^ c. |  | M | Italy | Italian | CT | DL |  | EuS |  | 0.0 |
| **Eu-1120** | 19-20^th^ c. |  | M | Italy | Italian | CT | DL |  | EuS |  | 0.0 |
| **Eu-1121** | 19-20^th^ c. |  | M | Italy | Italian | CT | DL |  | EuS |  | 0.0 |
| **Eu.24.00.1** | 19-20^th^ c. |  | M | France | French | CT | DL |  | EuS |  | 0.0 |
| **Eu.42.00.1** | 19-20^th^ c. |  | F | Italy | Italian | CT | DL |  | EuS |  | 0.0 |
| **Eu.42.00.2** | 19-20^th^ c. |  | M | Italy | Italian | CT | DL |  | EuS |  | 0.0 |
| **Eu.43.00.4** | 19-20^th^ c. |  | F | Malta | Maltese | CT | DL |  | EuS | 0.27 | 0.4 |
| **Total** |  |  |  |  |  |  |  |  |  | 0.39 |  |

^µ^3D models were obtained through the use of medical computed tomographic scans (CT), 3D surface scanning with an optical scanner (OP) and photogrammetry (PH).

*ACB: Academy of Science (Bucharest, Romania); Brno: Anthropos Institute (Brno, Czech Republic); DL: Duckworth Laboratory (Cambridge, United-Kingdom); DRACNA: Direction régionale des affaires culturelles (DRAC) Nouvelle-Aquitaine (Bordeaux, France); IEA: Institute of Ethnology and Anthropology RAS (Leninsky, Moscow, Russia); MAAP: Musée d'art et d'Archéologie du Périgord (Périgueux, France); MFP: Museo Fiorentino di Preistoria (Firenze, Italy); MH: Musée de l'Homme (Paris, France); MHN: Muséum d’Histoire naturelle Victor Brun (Montauban, France); MPG: Museo di Paleontologia e Geologia G. G. Gemmellaro, Universita degli Studi di Palermo (Palermo, Italy); MNP: Musée national de Préhistoire (Les Eyzies, France); NM: Naturhistorisches Museum (Vienna, Austrai); SAG: Soprintendenza Archeologia, Belle Arti e Paesaggio per la città metropolitana di Genova e le province di Imperia La Spezia e Savona (Genova, Italy); SAV: Soprintendenza Archeologia, Belle Arti e Paesaggio per l’area metropolitana di Venezia e le province di Belluno, Padova e Treviso (Venice, Italy).

1. **Raw data**

See, excel file Table S2.

1. **Supplementary analyses and results**


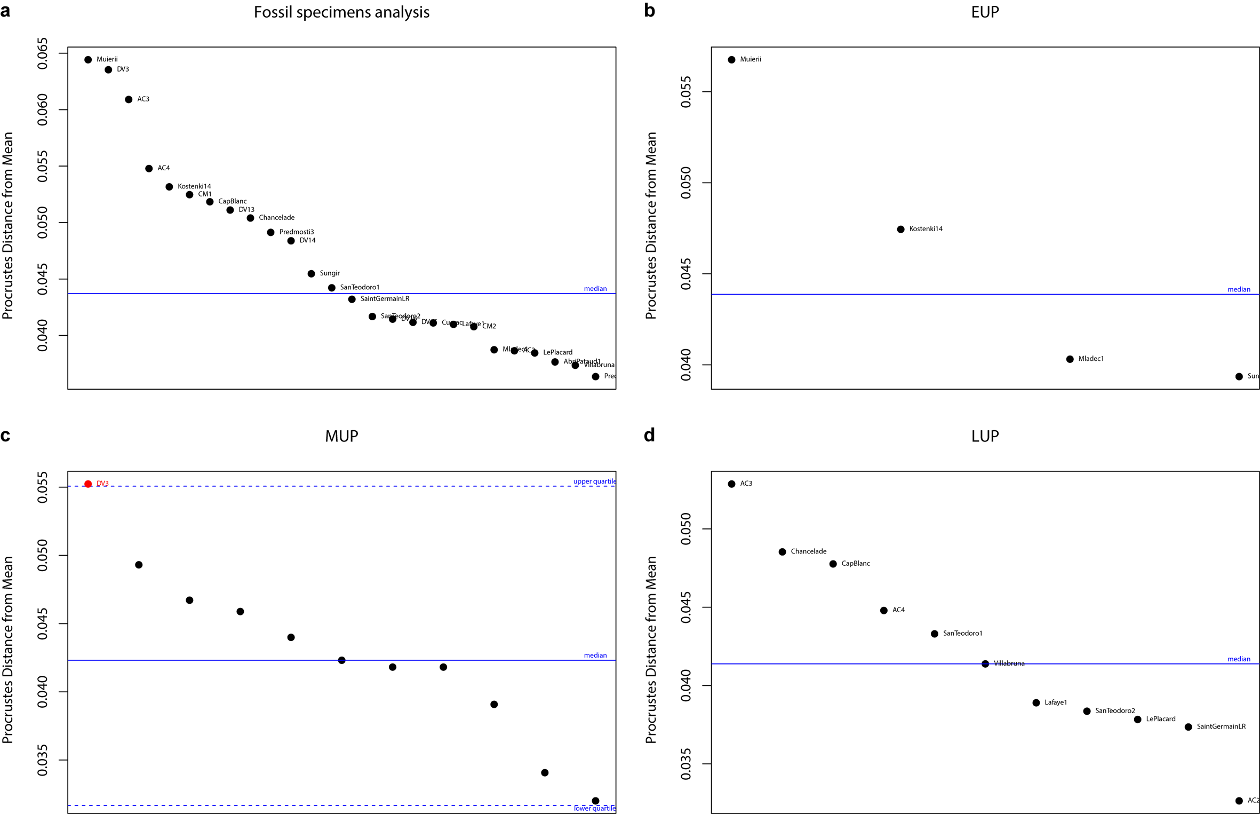


**Figure S6.** Outliers plot from PCA on the full cranium. The specimens are plotted according to their Procrustes distance to the mean shape of the data: a) Full dataset; b) EUP specimens only; c) MUP specimens only; d) LUP specimens only. There is no outlier when considering the full dataset, however, DV3 is outside of the normal range of variation when considering the MUP specimens only.

**
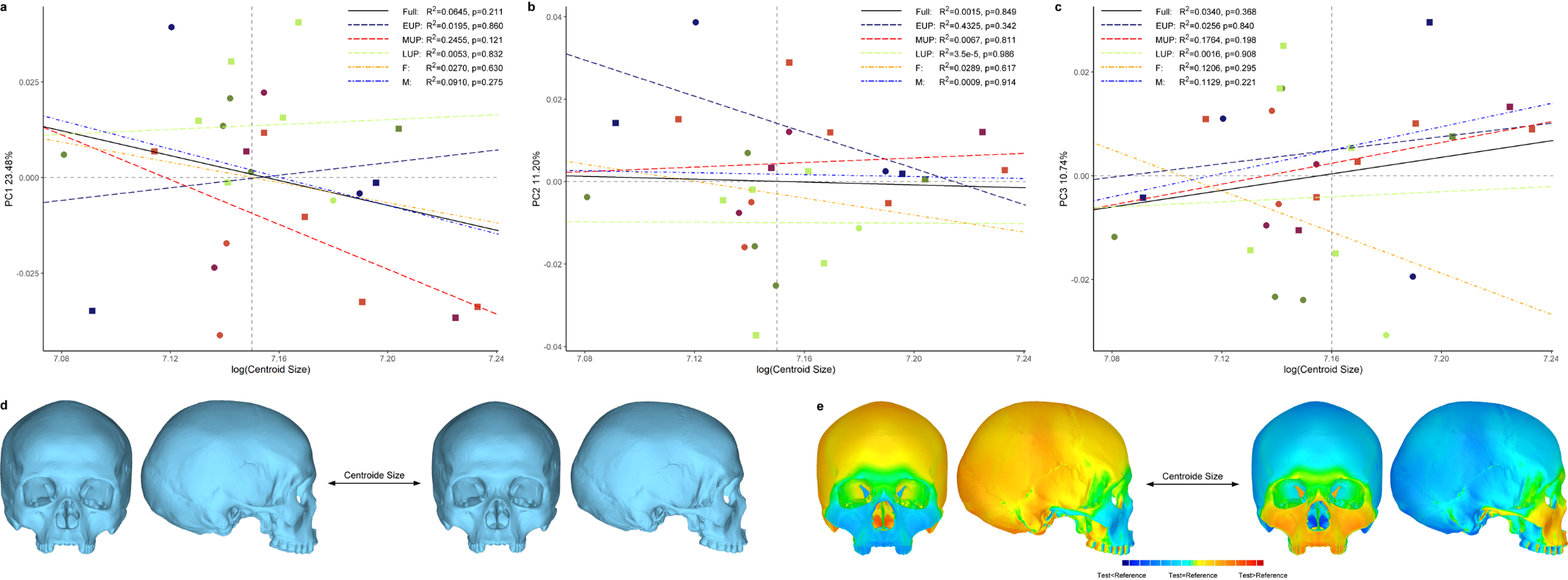
**

**Figure S7.** Linear regressions of size (log(centroid size)) against shape: a) PC1, b) PC2 and c) PC3. Five analyses were led on the full data (black), the three fossil samples (dark blue: EUP, red: MUP and green: LUP) and according to sex (orange: female, and blue: male).
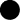
 represents female specimens;
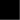
 represents male specimens. None of the regressions are significant, indicating that the absence of strong allometry in the data. d –warping and e –surface deviation spectrum) present the shape changes between small, which tends to have larger braincase and smaller faces, and large specimens.


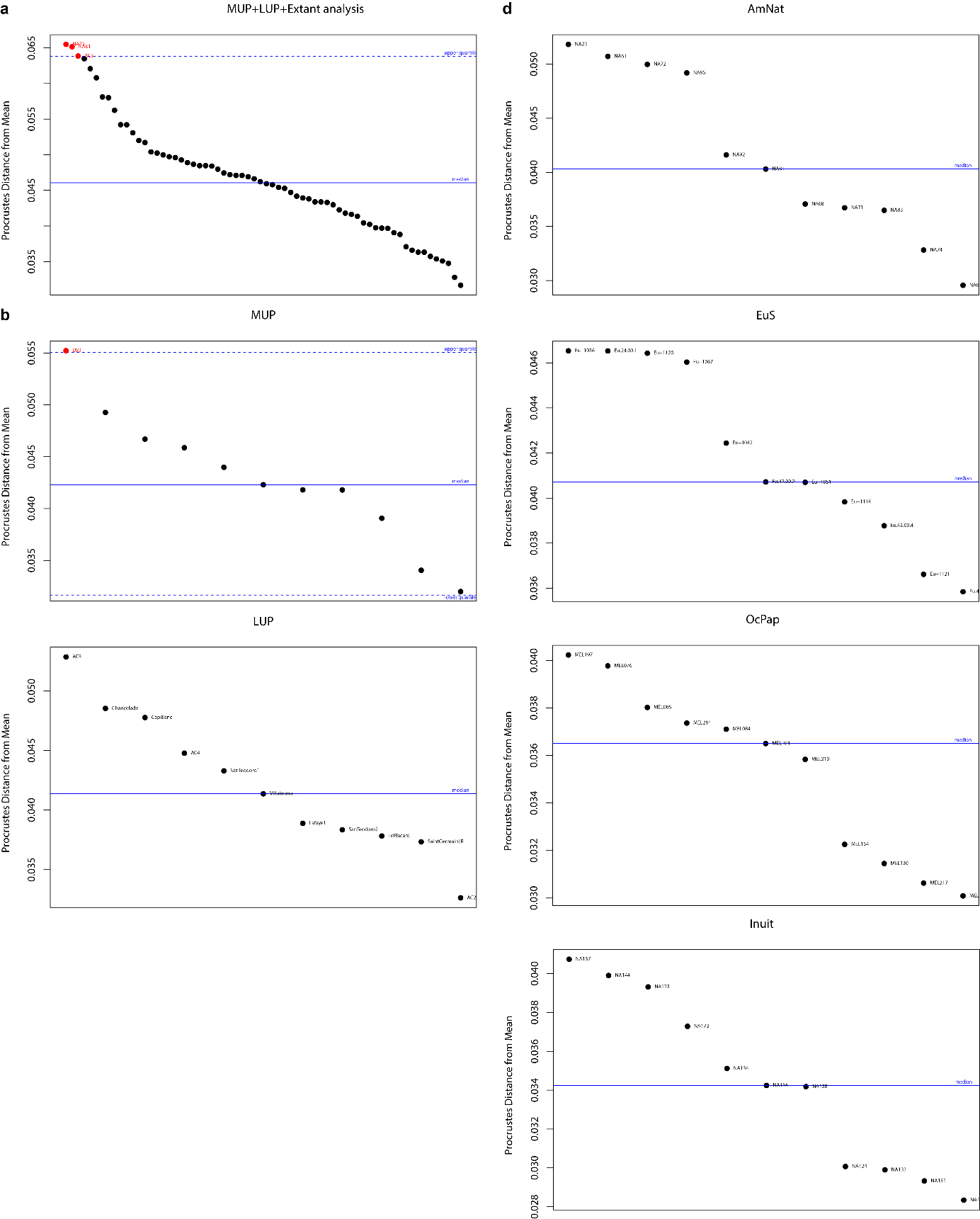


**Figure S8.** Outliers plot from PCA on the full cranium for the variance analysis. The specimens are plotted according to their Procrustes distance to the mean shape of the data: a) Full dataset, including the MUP, LUP and extant human samples; b) MUP and LUP specimens only; c) Extant populations: Native North American (AmNat), South Europeans (EuS), Papuans (OcPap) and Greenland Inuit (Inuit). There are three outliers when the full dataset is considered together (i.e. NA61, 72 and AC3), however, and to the exception of DV3 which is slightly outside of the variation of the MUP sample, when each sample is considered alone, there is no outlier.


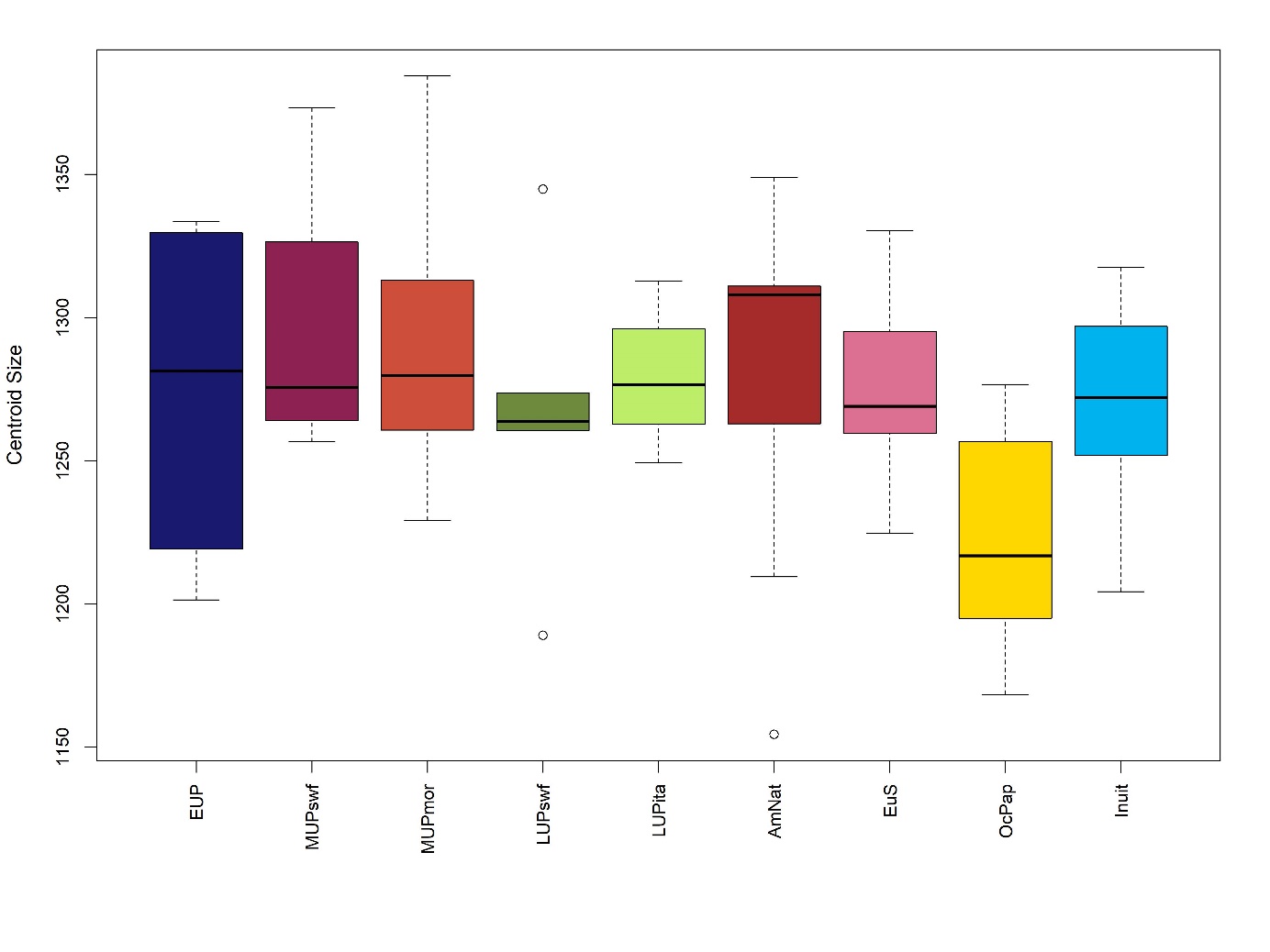
**Figure S9.** Centroid size of each specimen per population.


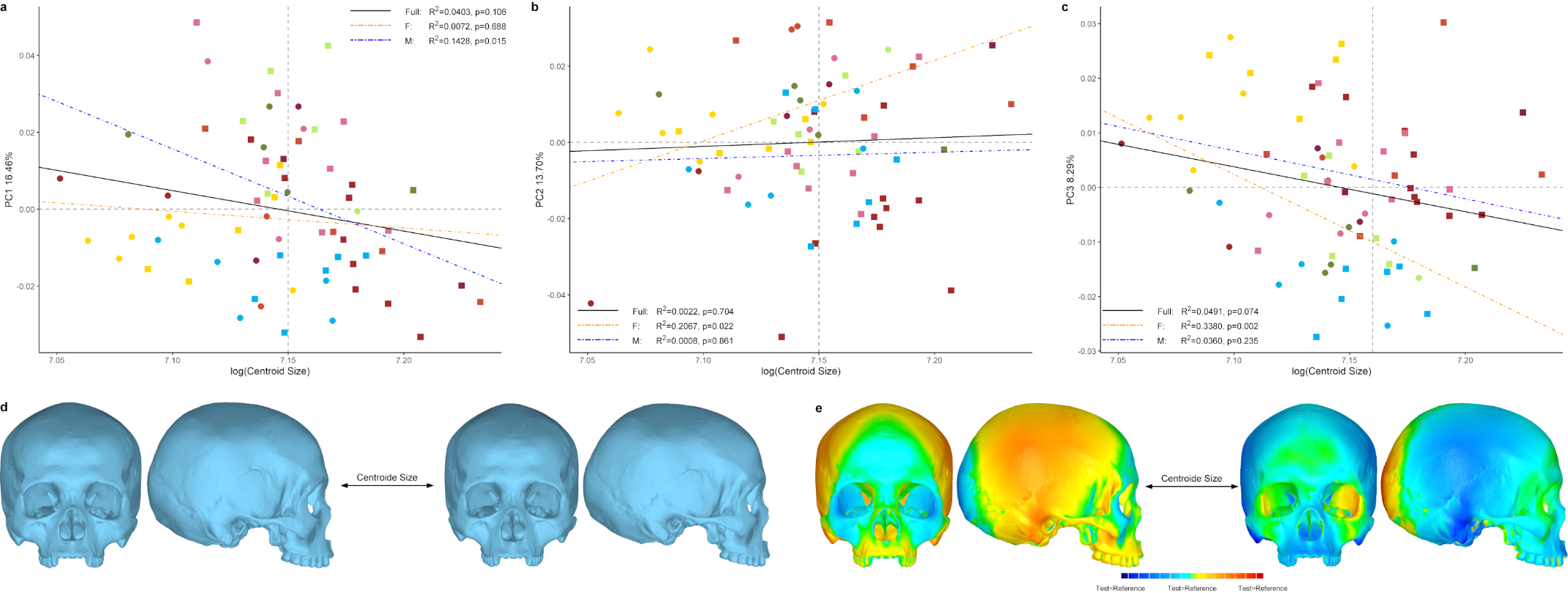


**Figure S10.** Linear regressions of size (log(centroid size)) against shape: a) PC1, b) PC2 and c) PC3 for the analysis of variance of the MUP, LU and extant human samples. Three analyses were led on the full data (black), and according to sex (orange: female, and blue: male).
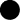
 represents female specimens;
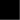
 represents male specimens. On PC1, size has a significant impact on shape when considering the male sample p=0.015). On PC2 the effect of size is significant for the female sample at p=0.022 and on PC3, it is significant for the female sample (p=0.002). d –warping and e –surface deviation spectrum) present the shape changes between small, which tends to have more rounded braincase and a more projecting lower face, and large specimens. The analyses of variance on the full cranium where done using the residuals from the regressions.

**Table S3.** PCA: Eigenvalues and variance explained by each PC of the analysis of the fossil sample. Allometry: R² and p value of the regressions of the shape (i.e. PC) on size (i.e. log(CS)) for the first 15 PCs

|  | **PCA** | | | **Allometry** | |
| --- | --- | --- | --- | --- | --- |
|  | **eigenvalues** | **% Variance** | **Cumulative %** | **R²** | **p** |
| **PC1** | 0.00054 | 23.48 | 23.48 | 0.0645 | 0.211 |
| **PC2** | 0.00026 | 11.20 | 34.69 | 0.0015 | 0.849 |
| **PC3** | 0.00025 | 10.74 | 45.43 | 0.0340 | 0.368 |
| **PC4** | 0.00022 | 9.35 | 54.78 | 0.0893 | 0.138 |
| **PC5** | 0.00017 | 7.21 | 62.00 | 0.0642 | 0.212 |
| **PC6** | 0.00013 | 5.43 | 67.43 | 0.1395 | 0.060 |
| **PC7** | 0.00010 | 4.52 | 71.95 | 0.0294 | 0.402 |
| **PC8** | 0.00008 | 3.61 | 75.56 | 0.0614 | 0.704 |
| **PC9** | 0.00007 | 3.21 | 78.76 | 0.0368 | 0.348 |
| **PC10** | 0.00006 | 2.71 | 81.48 | 0.0208 | 0.482 |
| **PC11** | 0.00006 | 2.61 | 84.09 | 0.1250 | 0.076 |
| **PC12** | 0.00005 | 2.32 | 86.40 | 0.0013 | 0.864 |
| **PC13** | 0.00005 | 2.04 | 88.45 | 0.0001 | 0.984 |
| **PC14** | 0.00004 | 1.64 | 90.09 | 0.0065 | 0.696 |
| **PC15** | 0.00004 | 1.55 | 91.64 | 0.0060 | 0.399 |
| **PC16** | 0.00003 | 1.41 | 93.05 | - | - |
| **PC17** | 0.00003 | 1.26 | 94.31 | - | - |
| **PC18** | 0.00003 | 1.16 | 95.47 | - | - |
| **PC19** | 0.00002 | 0.99 | 96.46 | - | - |
| **PC20** | 0.00002 | 0.79 | 97.25 | - | - |
| **PC21** | 0.00002 | 0.76 | 98.02 | - | - |
| **PC22** | 0.00002 | 0.65 | 98.67 | - | - |
| **PC23** | 0.00001 | 0.53 | 99.19 | - | - |
| **PC24** | 0.00001 | 0.47 | 99.67 | - | - |
| **PC25** | 0.00001 | 0.33 | 100.00 | - | - |

**Table S4.** Eigenvalues and variance explained by each BgPC of the analysis of the fossil sample.

|  | **eigenvalues** | **% Variance** | **Cumulative %** |
| --- | --- | --- | --- |
| **BgPC1** | 0.00040 | 77.66 | 77.66 |
| **BgPC2** | 0.00011 | 22.34 | 100.00 |

Results based on the discrimination of three groups: EUP, MUP and LUP.

**Table S5.** Eigenvalues and variance explained by each BgPC of analysis of the fossil sample.

|  | **eigenvalues** | **% Variance** | **Cumulative %** |
| --- | --- | --- | --- |
| **BgPC1** | 0.00032 | 55.02 | 55.02 |
| **BgPC2** | 0.00013 | 21.20 | 76.22 |
| **BgPC3** | 0.00010 | 16.16 | 92.38 |
| **BgPC4** | 0.00005 | 7.62 | 100.00 |

Results based on the discrimination of five groups: EUP, MUPCz, MUPFr, LUPeg and LUPmd.

**Table S6.** Eigenvalues and variance explained by each of the 30 first PCs of the analysis of the MUP, LUP and extant human samples.

|  | **PCA** | | | **Allometry** | |
| --- | --- | --- | --- | --- | --- |
|  | **eigenvalues** | **% Variance** | **Cumulative %** | **R²** | ***p*** |
| **PC1** | 0.00037 | 16.46 | 16.46 | 0.0403 | 0.106 |
| **PC2** | 0.00031 | 13.70 | 30.16 | 0.0022 | 0.704 |
| **PC3** | 0.00019 | 8.29 | 38.45 | 0.0491 | 0.074 |
| **PC4** | 0.00016 | 7.21 | 45.66 | 0.0244 | 0.211 |
| **PC5** | 0.00015 | 6.76 | 52.42 | 0.0362 | 0.126 |
| **PC6** | 0.00013 | 5.58 | 58.00 | 0.0071 | 0.501 |
| **PC7** | 0.00011 | 4.82 | 62.82 | - | - |
| **PC8** | 0.00009 | 3.95 | 66.77 | - | - |
| **PC9** | 0.00007 | 3.29 | 70.06 | - | - |
| **PC10** | 0.00006 | 2.53 | 72.59 | - | - |
| **PC11** | 0.00005 | 2.30 | 74.88 | - | - |
| **PC12** | 0.00005 | 2.07 | 76.96 | - | - |
| **PC13** | 0.00004 | 1.87 | 78.83 | - | - |
| **PC14** | 0.00004 | 1.69 | 80.52 | - | - |
| **PC15** | 0.00004 | 1.58 | 82.10 | - | - |
| **PC16** | 0.00003 | 1.40 | 83.49 | - | - |
| **PC17** | 0.00003 | 1.22 | 84.71 | - | - |
| **PC18** | 0.00003 | 1.21 | 85.93 | - | - |
| **PC19** | 0.00002 | 1.09 | 87.01 | - | - |
| **PC20** | 0.00002 | 0.95 | 87.97 | - | - |
| **PC21** | 0.00002 | 0.94 | 88.91 | - | - |
| **PC22** | 0.00002 | 0.82 | 89.73 | - | - |
| **PC23** | 0.00002 | 0.70 | 90.43 | - | - |
| **PC24** | 0.00001 | 0.66 | 91.09 | - | - |
| **PC25** | 0.00001 | 0.62 | 91.71 | - | - |
| **PC26** | 0.00001 | 0.56 | 92.27 | - | - |
| **PC27** | 0.00001 | 0.52 | 92.79 | - | - |
| **PC28** | 0.00001 | 0.49 | 93.27 | - | - |
| **PC29** | 0.00001 | 0.48 | 93.75 | - | - |
| **PC30** | 0.00001 | 0.43 | 94.19 | - | - |

**Table S7.** Eigenvalues and variance explained by each of the 30 first PCs of the analysis of the MUP, LUP and extant human samples after the exclusion of the outlier DV3.

|  | **eigenvalues** | **% Variance** | **Cumulative %** |
| --- | --- | --- | --- |
| **PC1** | 0.00037 | 16.61 | 16.61 |
| **PC2** | 0.00030 | 13.51 | 30.11 |
| **PC3** | 0.00019 | 8.52 | 38.63 |
| **PC4** | 0.00017 | 7.42 | 46.05 |
| **PC5** | 0.00015 | 6.77 | 52.82 |
| **PC6** | 0.00012 | 5.27 | 58.09 |
| **PC7** | 0.00011 | 4.95 | 63.04 |
| **PC8** | 0.00009 | 3.98 | 67.02 |
| **PC9** | 0.00007 | 3.36 | 70.37 |
| **PC10** | 0.00005 | 2.46 | 72.84 |
| **PC11** | 0.00005 | 2.15 | 74.98 |
| **PC12** | 0.00004 | 1.92 | 76.90 |
| **PC13** | 0.00004 | 1.85 | 78.75 |
| **PC14** | 0.00004 | 1.73 | 80.48 |
| **PC15** | 0.00003 | 1.56 | 82.04 |
| **PC16** | 0.00003 | 1.42 | 83.46 |
| **PC17** | 0.00003 | 1.25 | 84.71 |
| **PC18** | 0.00003 | 1.21 | 85.92 |
| **PC19** | 0.00002 | 1.12 | 87.04 |
| **PC20** | 0.00002 | 0.98 | 88.02 |
| **PC21** | 0.00002 | 0.92 | 88.94 |
| **PC22** | 0.00002 | 0.80 | 89.74 |
| **PC23** | 0.00002 | 0.71 | 90.45 |
| **PC24** | 0.00002 | 0.67 | 91.12 |
| **PC25** | 0.00001 | 0.61 | 91.73 |
| **PC26** | 0.00001 | 0.57 | 92.31 |
| **PC27** | 0.00001 | 0.51 | 92.81 |
| **PC28** | 0.00001 | 0.50 | 93.31 |
| **PC29** | 0.00001 | 0.49 | 93.79 |
| **PC30** | 0.00001 | 0.44 | 94.24 |

**References**

1. Bronk Ramsey, C. Bayesian Analysis of Radiocarbon Dates. *2009* **51**, 337-360 (2009).
2. Reimer, P. J. *et al.* IntCal13 and Marine13 Radiocarbon Age Calibration Curves 0–50,000 Years cal BP. *Radiocarbon* **55** (2013).
3. Trinkaus, E. & Buzhilova, A. P. Diversity and differential disposal of the dead at Sunghir. *Antiquity* **92**, 7-21, doi:10.15184/aqy.2017.223 (2018).
4. Dinnis, R. *et al.* The Age of the ‘Anosovka-Tel’manskaya Culture’ and the Issue of a Late Streletskian at Kostёnki 11, SW Russia. *Proc. Prehist. Soc.* **84**, 21-40, doi:10.1017/ppr.2018.1 (2018).
5. Bradley, B. A., Anikovich, M. & Giria, E. Early Upper Palaeolithic in the Russian Plain: Streletskayan flaked stone artefacts and technology. *Antiquity* **69**, 989-998, doi:10.1017/S0003598X00082521 (1995).
6. Chabai, V. in *The Chronology of the Aurignacian and of the Transitional Technocomplexes: Dating, Stratigraphies, Cultural Implications. .* (eds J. Zilhao & A. d'Errico) 71-86 (Instituto Português de Arqueologia).
7. Matyukhin, A. E. & Sapelko, T. V. PALEOLITHIC SITE BIRYUCHYA BALKA-2: GEOLOGY, CHRONOLOGY, AND PALEOECOLOGY. *Archaeology, Ethnology and Anthropology of Eurasia* **37**, 2-12, doi:https://doi.org/10.1016/j.aeae.2010.02.004 (2009).
8. Svendsen, J. I. *et al.* Geo-archaeological investigations of Palaeolithic sites along the Ural Mountains – On the northern presence of humans during the last Ice Age. *Quaternary Science Reviews* **29**, 3138-3156, doi:https://doi.org/10.1016/j.quascirev.2010.06.043 (2010).
9. Gavrilov, K. N. in *Le Sungirien* Vol. 147 (eds Sergey V. Vasilyev, A. Sinitsyn, & M. Otte) 107-117 (ERAUL, 2017).
10. Villotte, S. *Enthésopathies et activités des hommes préhistoriques Recherche méthodologique et application aux fossiles européens du Paléolithique supérieur et du Mésolithique*. Vol. 1992 (Archaeopress, 2009).
11. Trinkaus, E., Buzhilova, A. P., Mednikova, M. B. & Dobrovolskaya, M. V. *The People of Sunghir. Burials, Bodies, and Behavior in the Earlier Upper Paleolithic.* . 340 (Oxford University Press, 2014).
12. Marom, A., McCullagh, J. S., Higham, T. F., Sinitsyn, A. A. & Hedges, R. E. Single amino acid radiocarbon dating of Upper Paleolithic modern humans. *Proc Natl Acad Sci U S A* **109**, 6878-6881, doi:10.1073/pnas.1116328109 (2012).
13. Nalawade-Chavan, S., McCullagh, J. & Hedges, R. New Hydroxyproline Radiocarbon Dates from Sungir, Russia, Confirm Early Mid Upper Palaeolithic Burials in Eurasia. *PLOS ONE* **9**, e76896, doi:10.1371/journal.pone.0076896 (2014).
14. Dinnis, R. *et al.* New data for the Early Upper Paleolithic of Kostenki (Russia). *J. Hum. Evol.* **127**, 21-40, doi:https://doi.org/10.1016/j.jhevol.2018.11.012 (2019).
15. Haesaerts, P., Damblon, F., Sinitsyn, A. & Van der Plicht, J. Kostienki 14 (Voronezh, Central Russia): new data on stratigraphy and radiocarbon chronology. *BAR International Series* **1240**, 169-180 (2004).
16. Sinitsyn, A. in *New aspects of the Central and Eastern European Upper Palaeolithic – methods, chronology, technology and subsistence, Prehistoric Commission of the Austrian Academy of Sciences, Vienna, November 9-11, 2005.* (eds C. Neugebauer-Maresch & L.R. Owen) 27-48 (Verlag der Österreichischen Akademie der Wissenschaften).
17. Sinitsyn, A. A. A Palaeolithic ‘Pompeii’ at Kostenki, Russia. *Antiquity* **77**, 9-14, doi:10.1017/S0003598X00061299 (2003).
18. Sinitsyn, A. A. & Hoffecker, J. F. Radiocarbon dating and chronology of the Early Upper Paleolithic at Kostenki. *Quatern. Int.* **152-153**, 164-174, doi:https://doi.org/10.1016/j.quaint.2005.12.007 (2006).
19. Sinitsyn, A. in *La spiritualité. Actes du colloque de la commission 8 de l'UISPP (Paléolithique supérieur), Liège, 10–12 décembre 2003.* (ed M. Otte) 237-244 (ERAUL).
20. Douka, K. & Higham, T. The Chronological Factor in Understanding the Middle and Upper Paleolithic of Eurasia. *Curr. Anthrop.* **58**, S480-S490, doi:10.1086/694173 (2017).
21. Svoboda, J. The depositional context of the Early Upper Paleolithic human fossils from the Koněprusy (Zlatý kůň) and Mladeč Caves, Czech Republic. *J. Hum. Evol.* **38**, 523-536, doi:https://doi.org/10.1006/jhev.1999.0361 (2000).
22. Wolpoff, M. H., Frayer, D. W. & Jelínek, J. in *Early Modern Humans at the Moravian Gate* (ed M. Teschler-Nicola) 273-340 (Springer, 2006).
23. Wild, E. M. *et al.* Direct dating of Early Upper Palaeolithic human remains from Mladec. *Nature* **435**, 332-335, doi:http://www.nature.com/nature/journal/v435/n7040/suppinfo/nature03585_S1.html (2005).
24. Doboş, A., Soficaru, A. & Trinkaus, E. y. a. p. o. t. P. M. R. *The prehistory and paleontology of the Peştera Muierii (Romania)*. Vol. 124 (ERAUL, 2012).
25. Soficaru, A., Doboş, A. & Trinkaus, E. Early modern humans from the Peştera Muierii, Baia de Fier, Romania. *Proceedings of the National Academy of Sciences* **103**, 17196-17201, doi:10.1073/pnas.0608443103 (2006).
26. Olariu, A. *et al.* in *Applications of High Precision Atomic and Nuclear Methods* (eds A. Olariu, K. Stenström, & K. Hellborg) 222-226 (Editura Academiei Romäne, 2005).
27. Svoboda, J., Klíma, B., Jarošová, L. & Škrdla, P. in *Hunters of the Golden Age* Vol. 31 *Analecta Praehistorica Leidensia* (eds W. Roebroeks, Margherita Mussi, Jiří Svoboda, & K. Fennema) 197-217 (Sidestone Press, 2000).
28. Trinkaus, E. & Svoboda, J. (Oxford University Press, New York, 2006).
29. Trinkaus, E. & Jelı́nek, J. Human remains from the Moravian Gravettian: the Dolnı́ Věstonice 3 postcrania. *J. Hum. Evol.* **33**, 33-82, doi:https://doi.org/10.1006/jhev.1997.0142 (1997).
30. Fewlass, H. *et al.* Direct radiocarbon dates of mid Upper Palaeolithic human remains from Dolní Věstonice II and Pavlov I, Czech Republic. *Journal of Archaeological Science: Reports* **27**, 102000, doi:https://doi.org/10.1016/j.jasrep.2019.102000 (2019).
31. Svoboda, A. J. The Upper Paleolithic burial area at Předmostí: ritual and taphonomy. *J. Hum. Evol.* **54**, 15-33, doi:http://dx.doi.org/10.1016/j.jhevol.2007.05.016 (2008).
32. Velemínská, J. & Bruzek, J. (Academia, Praha, 2008).
33. Matiegka, J. *Homo předmostensis, fosilní člověk z Předmostí na Moravě. T1. Nákladem České akademie věd a umění.*, 176 (V. Praze, 1934).
34. Lartet, L. Une sépulture des troglodytes du Périgord (crânes des Eyzies). *Bull. Mém. Soc. Anthrop. Paris* **II**, 335-349, doi:https://doi.org/10.3406/bmsap.1868.9547 (1868).
35. Henry-Gambier, D., Nespoulet, R. & Chiotti, L. An Early Gravettian cultural attribution for the human fossils from the Cro-Magnon rock shelter (Les Eyzies-de-Tayac, Dordogne). *Paleo* **24**, 121-138 (2013).
36. Partiot, C., Trinkaus, E., Knüsel, C. J. & Villotte, S. The Cro-Magnon babies: Morphology and mortuary implications of the Cro-Magnon immature remains. *Journal of Archaeological Science: Reports* **30**, 102257, doi:https://doi.org/10.1016/j.jasrep.2020.102257 (2020).
37. Villotte, S. & Balzeau, A. Que reste-t-il des Hommes de Cro-Magnon 150 ans après leur découverte ? *BMSAP* **30**, 146-152 (2018).
38. Thibeault, A. & Villotte, S. Disentangling Cro-Magnon: A multiproxy approach to reassociate lower limb skeletal remains and to determine the biological profiles of the adult individuals. *Journal of Archaeological Science: Reports* **21**, 76-86, doi:https://doi.org/10.1016/j.jasrep.2018.06.038 (2018).
39. Henry-Gambier, D. Les fossiles de Cro-Magnon (Les Eyzies-de-Tayac, Dordogne) : nouvelles données sur leur position chronologique et leur attribution culturelle. *Bull. Mém. Soc. Anthrop. Paris* **14**, 89-112 (2002).
40. Guyomarc'h, P. *et al.* New data on the paleobiology of the Gravettian individual L2A from Cussac cave (Dordogne, France) through a virtual approach. *Journal of Archaeological Science: Reports* **14**, 365-373, doi:https://doi.org/10.1016/j.jasrep.2017.06.005 (2017).
41. Kacki, S. *et al.* Complex mortuary dynamics in the Upper Paleolithic of the decorated Grotte de Cussac, France. *Proceedings of the National Academy of Sciences*, 202005242, doi:10.1073/pnas.2005242117 (2020).
42. Jaubert, J. *et al.* The chronology of human and animal presence in the decorated and sepulchral cave of Cussac (France). *Quatern. Int.* **432**, 5-24, doi:https://doi.org/10.1016/j.quaint.2016.01.052 (2017).
43. Villotte, S., Santos, F. & Courtaud, P. In situ study of the Gravettian individual from Cussac cave, locus 2 (Dordogne, France). *Am. J. Phys. Anthropol.* **158**, 759-768, doi:10.1002/ajpa.22831 (2015).
44. Douka, K., Chiotti, L., Nespoulet, R. & Higham, T. A refined chronology for the Gravettian sequence of Abri Pataud. *J. Hum. Evol.* **141**, 102730, doi:https://doi.org/10.1016/j.jhevol.2019.102730 (2020).
45. Billy, G. in *Excavation of the Abri Pataud. Les Eyzies (Dordogne)* Vol. 30 (ed H.L. Movius) 201-261 (Harvard University, Peabody Museum, American School of Prehistoric Research, 1975).
46. Henry-Gambier, D., Villotte, S., Beauval, C., Brůžek, J. & Grimaud-Herve, D. in *Le Gravettien final de l’abri Pataud (Dordogne, France). Fouilles et études 2005-2009* Vol. 2458 *BAR International Series* (eds R. Nespoulet, L. Chiotti, & D. Henry-Gambier) 135-177 (Archaeopress, 2013).
47. Villotte, S., Chiotti, L., Nespoulet, R. & Henry-Gambier, D. Anthropological study of recently discovered human remains from the layer 2 of the abri Pataud (Les Eyzies-de-Tayac-Sireuil, Dordogne, France). *BMSAP* **27**, 158-188, doi:10.1007/s13219-015-0128-3 (2015).
48. Testut, L. Recherches anthropologiques sur le squelette quaternaire de Chancelade (Dordogne). *Publications de la Société Linnéenne de Lyon* **8**, 131-246, doi:https://doi.org/10.3406/linly.1889.16318 (1889).
49. Hardy, M. in *Congrès international d'anthropologie et d'archéologie préhistorique. Comptes-rendus de la 10ème session.* 398-404.
50. Vallois, H. V. Nouvelles recherches sur l'Homme de Chancelade. *L'Anthropologie* **50**, 165-202 (1941).
51. Barshay-Szmidt, C. *et al.* New extensive focused AMS 14C dating of the Middle and Upper Magdalenian of the western Aquitaine/Pyrenean region of France (ca. 19–14 ka cal BP): Proposing a new model for its chronological phases and for the timing of occupation. *Quatern. Int.* **414**, 62-91, doi:http://dx.doi.org/10.1016/j.quaint.2015.12.073 (2016).
52. Sécher, A. *Traditions techniques et paléogéographie du Magdalénien moyen ancien dans le Sud-Ouest de la France (19 000 – 17 500 cal. BP). Des groupes humains à plusieurs visages ?* Ph.D. thesis, Université de Bordeaux, (2017).
53. Dastugue, J. & de Lumley, M. A. in *La préhistoire française* (ed H. de Lumley) 612-622 (CNRS éditions, 1976).
54. Guéret, M. La station Magdalénienne de Bruniquel. *Bulletin de la Société de Sciences Naturelles du Tarn et Garonne* **1**, 26-27 (1952).
55. Gambier, D., Valladas, H., Tisnérat-Laborde, N., Arnold, M. & Bresson, F. Datation de vestiges humains présumés du Paléolithique supérieur par la méthode du Carbone 14 en spectrométrie de masse par accélérateur. *Paleo* **12**, 201-212 (2000).
56. Vanhaeren, M. & d’Errico, F. Grave goods from the Saint-Germain-la-Rivière burial: Evidence for social inequality in the Upper Palaeolithic. *J. Anthrop. Archaeol.* **24**, 117-134, doi:https://doi.org/10.1016/j.jaa.2005.01.001 (2005).
57. Blanchard, R. Découverte d’un squelette humain à Saint-Germain-la-Rivière. *Revue historique et archéologique du Libournais* **9**, 11-18 (1935).
58. Henry-Gambier, D., Bruzek, J., Murail, P. & Houët, F. Révision du sexe du squelette magdalénien de Saint-Germain-la-Rivère (Gironde, France). *PALEO* **14**, 205-212 (2002).
59. Lalanne, J.-G. & Breuil, H. L’abri sculpté de Cap-Blanc à Laussel (Dordogne). *L'Anthropologie* **XXII**, 385-402 (1911).
60. Bourdier, C. Paléogéographie symbolique au Magdalénien moyen. Apport de l'étude des productions graphiques pariétales des abris occupés et sculptés de l'Ouest français (Roc-aux-Sorciers, Chaire-à-Calvin, Reverdit, Cap-Blanc). *Bulletin de la Société préhistorique française* **109**, 346-348 (2012).
61. Bourdier, C., Pétillon, J.-M., Chehmana, L. & Valladas, H. in *Actes du colloque « Micro-analyses et datations de l'art préhistorique dans son contexte archéologique », MADAPCA - Paris, 16-18 novembre 2011* (ed Patrick Paillet) 285-294 (PALEO, 2014).
62. Boulestin, B. & Henry-Gambier, D. *Les restes humains badegouliens de la Grotte du Placard. Cannibalisme et guerre il y a 20,000 ans*. (Archaeopress Archaeology, 2019).
63. Hamy, J. in *Congrès international d'anthropologie et d'archéologie préhistoriques.* 405-450 (Leroux).
64. Sparacello, V. *et al.* New insights on Final Epigravettian funerary behavior at Arene Candide Cave (Western Liguria, Italy). *Journal of Anthropological Sciences* **96**, doi:10.4436/jass.96003 (2018).
65. Bietti, A. Some remarks on the new radiocarbon dates from the Arene Candide Cave (Savona, Italy). *Hum. Evol.* **2**, 185-190, doi:10.1007/BF02436406 (1987).
66. Formicola, V., Pettitt, P. B., Maggi, R. & Hedges, R. Tempo and mode of formation of the Late Epigravettian necropolis of Arene Candide cave (Italy): direct radiocarbon evidence. *J. Archaeol. Sci.* **32**, 1598-1602, doi:https://doi.org/10.1016/j.jas.2005.04.013 (2005).
67. Paoli, G., Parenti, R. & Sergi, S. Gli scheletri mesolitici della caverna delle Arene Candide (Liguria). *Memorie dell’Istituto Italiano di Paleontologia Umana* **3**, 33-154 (1980).
68. Vercellotti, G., Alciati, G., Richards, M. P. & Formicola, V. The Late Upper Paleolithic skeleton Villabruna 1 (Italy): a source of data on biology and behavior of a 14,000 year-old hunter. *Journal of Anthropological Sciences* **86**, 143-163 (2008).
69. Broglio, A. in *Actes Du Colloque International de Liège.* (ed M. Otte) 847-869 (ERAUL).
70. Mannino, M. A. *et al.* Upper Palaeolithic hunter-gatherer subsistence in Mediterranean coastal environments: an isotopic study of the diets of the earliest directly-dated humans from Sicily. *J. Archaeol. Sci.* **38**, 3094-3100, doi:https://doi.org/10.1016/j.jas.2011.07.009 (2011).
71. Martini, F. *et al.* in *Cosmopolitismo e Regionalità Nel Tardoglaciale* (ed EDIFIR-Edizioni Firenze) 209-254 (2007).
72. D'Amore, G., Di Marco, S., Tartarelli, G., Bigazzi, R. & Sineo, L. Late Pleistocene human evolution in Sicily: comparative morphometric analysis of Grotta di San Teodoro craniofacial remains. *J. Hum. Evol.* **56**, 537-550 (2009).
73. Fabbri, P. F. Nuove determinazioni del sesso e della statura degli individui 1 e 4 del Paleolitico superiore della Grotta di San Teodoro. *Rivista di scienze preistoriche* **45**, 219-232 (1993).
74. Samsel, M. *Microévolution et bioarchéologie des groupes humains de la fin du Pléistocène et du début de l’Holocène en Europe occidentale : apports de l’anthropologie biologique aux connaissances sur le Paléolithique final et le Mésolithique* Ph.D. thesis, Université de Bordeaux, (2018).
75. Messina, A., Miccichè, R., Di Lorenzo, D., Carotenuto, G. & Sineo, L. A Revised Sex Assessment of the Epigravettian Human Remain ST1 from San Teodoro Cave (Messina, Sicily). *Archivio per l’Antropologia e la Etnologia* **143**, 115-123 (2013).
